# Narcolepsy with cataplexy: Does age at diagnosis change the clinical picture?

**DOI:** 10.1111/cns.13438

**Published:** 2020-08-06

**Authors:** Min Zhang, Clara Odilia Inocente, Carine Villanueva, Michel Lecendreux, Yves Dauvilliers, Jian‐Sheng Lin, Isabelle Arnulf, Marie‐Paule Gustin, Marine Thieux, Patricia Franco

**Affiliations:** ^1^ Integrative Physiology of the Brain Arousal Systems CRNL INSERM‐U1028 CNRS UMR5292 University of Lyon 1 Lyon France; ^2^ Endocrinology Pediatric Unit Woman Mother Child Hospital Civil Hospices of Lyon Lyon France; ^3^ Pediatric Sleep Centre Hospital Robert‐Debre Paris France; ^4^ National Reference Centre for Orphan Diseases Narcolepsy, Idiopathic Hypersomnia, and Kleine‐Levin Syndrome Paris France; ^5^ National Reference Network for Narcolepsy Sleep‐Wake Disorder Unit Department of Neurology Gui‐de‐Chauliac Hospital CHU Montpellier Montpellier France; ^6^ Inserm U1061 University of Montpellier Neuropsychiatry: Epidemiological and Clinical Research Montpellier France; ^7^ AP‐HP Pitié‐Salpêtrière Hospital Sleep Disorder Unit & Sorbonne University Paris France; ^8^ Emerging Pathogens Laboratory–Fondation Mérieux International Center for Infectiology Research (CIRI) Inserm U1111 CNRS UMR5308 ENS de Lyon Lyon France; ^9^ Institute of Pharmaceutic and Biological Sciences Public Health Department Biostatistics University Claude Bernard Lyon 1 Villeurbanne France; ^10^ Sleep Pediatric Unit Woman Mother Child Hospital Civil Hospices of Lyon Lyon France

**Keywords:** cataplexy, children, Narcolepsy, obesity, sleep

## Abstract

**Objective:**

To compare symptoms and sleep characteristics in patients diagnosed with narcolepsy‐cataplexy (NC) before and after the age of 18 years.

**Methods:**

De novo patients with NC diagnosis completed a standardized questionnaire and interview, followed by a sleep study. The clinical and sleep measures were compared between patients diagnosed before (46 children, median age: 12 year old) and after (46 adults, median age: 28.5 year old) 18 years of age.

**Results:**

The frequency of obesity (54% vs 17%), night eating (29% vs 7%), parasomnia (89% vs 43%), sleep talking (80% vs 34%), and sleep drunkenness (69% vs 24%) were higher in children than in adults, the frequency of sleep paralysis was lower (20% vs 55%) but the frequency of cataplexy and the severity of sleepiness were not different. Children scored higher than adults at the attention‐deficit/hyperactivity disorder (ADHD) scale. Depressive feelings affected not differently children (24%) and adults (32%). However, adults had lower quality of life than children. There was no difference between groups for insomnia and fatigue scores. Quality of life was essentially impacted by depressive feelings in both children and adults. Obstructive apnea‐hypopnea index (OAHI) was lower in children with higher mean and minimal oxygen saturation than in adults. No between‐group differences were found at the multiple sleep latency test. The body mass index (*z*‐score) was correlated with OAHI (*r* = .32).

**Conclusion:**

At time of NC diagnosis, children have more frequent obesity, night eating, parasomnia, sleep talking, drunkenness, and ADHD symptoms than adults, even if sleepiness and cataplexy do not differ. These differences should be considered to ensure a prompt diagnosis.

## Graphical abstract

At time of diagnosis, children and adults suffering from narcolepsy with cataplexy present different clinical and sleep characteristics. Narcoleptic children have more frequent obesity, parasomnia, sleep talking, drunkenness, and ADHD symptoms than adults. Narcoleptic adults suffer more frequently from OSA and have lower quality of life than narcoleptic children. These differences should be considered to ensure a prompt diagnosis.

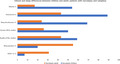

## 1 INTRODUCTION

Narcolepsy with cataplexy (NC) is a rare neurological disorder characterized by excessive daytime sleepiness (EDS) with irresistible sleep attacks and cataplexy (sudden loss of muscle tone triggered by emotions), associated sometimes with other abnormal rapid eye movement (REM) manifestations such as hypnagogic hallucinations, sleep paralysis, REM sleep behavior disorder (RBD), and disturbed nocturnal sleep.^1^ The frequent comorbidities of NC include obesity, restless legs syndrome (RLS), periodic limb movement syndrome (PLMS), depressive mood, and symptoms of attention‐deficit/hyperactivity disorder (ADHD). The prevalence of narcolepsy ranges from 0.02% to 0.05% in European and North American populations.^2^, ^3^ The NC prevalence increased after the H1N1 infection and vaccine.^4^, ^5^ Narcolepsy with cataplexy is caused by a loss of hypocretin‐1 neurons located in the lateral hypothalamus,^6^ probably via an autoimmune mechanism.^7^, ^8^ Indeed, low cerebrospinal fluid (CSF) hypocretin‐1 levels (less than or equal to 110 pg/mL) are found in more than 90% of patients with NC (also called narcolepsy type 1 or hypocretin deficient). In opposite, in narcolepsy type 2, there is no decrease in hypocretin levels. Additionally, the human leukocyte antigen (HLA) DQB1*0602 genotype is closely associated with NC.^9^

The question of age at onset and age at diagnosis is important in the context of NC. Despite more than half of patients with NC have a disease onset prior to the age of 18 years,^10^ there is a delay of more than 10 years between the disease onset and its diagnosis.^11^ Indeed, many people with an NC onset during childhood or adolescence are diagnosed only when they are adults, after they completed and possibly failed their academic studies, gained an almost irreversible obesity and experienced low self‐esteem. There may be several reasons for this delay, including the rarity of NC, reduced awareness about this disorder among clinicians, but also changes in the clinical picture of NC depending on age at diagnosis. Indeed, Aran et al reported that children with postpubertal onset of narcolepsy had a higher prevalence of sleep paralysis and hypnagogic hallucinations, and shorter mean sleep latency (MSL) than children with peripubertal and prepubertal onset.^12^ In large groups, there was a decrease in the number of sleep onset rapid eye movement periods (SOREMPs) and an increase of MSL with age gain, with no difference across age in terms of clinical complaints.^10^, ^13^

Comorbidities may also vary as a result of age in NC. Obesity in NC affects more than 50% of children^14^, ^15^ and only 30% of adults.^16^, ^17^ Aran et al^12^ found increased PLMs in children with postpubertal NC onset but not in younger patients. Nevsimalova et al^18^ reported that the most frequent comorbidities occurred in the oldest (>60 year) group compared to patients younger than 20 years, including obstructive sleep apnea (OSA) (53% vs 0%), RBD (40% vs 32%), PLMs (34% vs 16%), and RLS (21% vs 0%). The authors noticed that cataplexy was more frequent in the adults (80%) than in children and adolescents (52%).^18^ Because these results were obtained in mixed groups (among these patients, 83% had NC and 17% had narcolepsy without cataplexy, which is a different form of narcolepsy), and could be secondary to treatments (76% were treated with stimulants or anti‐cataplexy medications at time of study) which could promote RBD, RLS, PLMs, and weight changes, there is a need for studying de novo, untreated patients in order to obtain a genuine picture.

There is a need for studying de novo, untreated patients in order to obtain a genuine picture. Therefore, we aimed at systematically characterizing the symptoms, signs, HLA positivity and sleep measures in nighttime polysomnography and multiple sleep latency tests (MSLT) at time of diagnosis in de novo (untreated) patients, and at comparing these measures according to the age at diagnosis (≥18 years or <18 years). In theory, it would be more interesting to evaluate patients at disease onset before and after the age of 18 years but patients often did not clearly remember the onset time of the first symptoms or did not have a complete investigation at this time. The aim of this study was to identify differences which could drive the attention of clinicians towards an earlier recognition of NC in children at the moment of the diagnosis.

## 2 METHODS

### 2.1 Patients

In this retrospective study, all children (age < 18 years old) and adults (age ≥ 18 years old) presenting in the 4 national reference centers for narcolepsy with newly diagnosed NC between 2008 and 2011 were proposed to take part in the research program NARCOBANK (PHRC AOM07‐138, principal investigator Isabelle Arnulf). Most patients (99.6%) agreed to take part in the program, which included a systematic interview of patients (and parents) with the physician in charge (ML, PF, YD, IA) and a collection of the results of sleep study and HLA genotyping. Both the adult patients or pediatric patients and their parents signed written consent forms. This study was approved by the ethics committee (Comité de Protection des Personnes Ile de France ‐ 6).

The cohort included 46 de novo patients diagnosed with NC before 18 years and 46 patients diagnosed with NC after 18 years. All the patients had clear‐cut cataplexy evaluated by sleep specialists. No patient was treated at time of diagnostic procedure.

### 2.2 Questionnaires

The severity of daytime sleepiness was evaluated in adults using the score at the *Epworth sleepiness scale* (ESS, range 0‐24)^19^ and in children using the score at the *adapted Epworth sleepiness score* (AESS), in which the item “falling asleep while in a car stopped in the traffic” was replaced to “falling asleep at school”.^20^ Scores greater than 10 were considered abnormal.

The severity of cataplexy was evaluated using the *Cataplexy Severity Rating Score* (1 = moderate weakness, for example, head drop or jaw opening; 2 = can maintain posture with external support; 3 = loses posture and falls to the ground).^21^ However, a patient can have different types of cataplexy (type 1, 2, 3). The frequency of cataplexy attacks was also evaluated using a Likert scale (0: less than one episode per year; 1: more than 1 attack per year; 2: more than 1 attack per month, 3: more than 1 episode per week; 4: more than 1 episode per day).^22^ For the frequency of partial cataplexy, there was no distinction between type 1 or type 2.

To assess depressive symptoms, the *Beck Depression Inventory (BDI)* was used in adults^23^ and the *Children's Depression Inventory* (CDI) in children,^24^ with scores greater than or equal to 16 considered as abnormal for both scales.

The symptoms associated with attention‐deficit/hyperactivity disorder were scored by adults with NC^25^ and by parents of children with NC using the *Conners Parents Rating Scale‐Revised (Conners RS‐R)*.^26^ Moderate to severe symptoms were defined with a cutoff above 65, severe symptoms with a cutoff above 75.

Fatigue was scored from 0 to 14 using the *Chalder's fatigue scale* ^27^ in adults and children, with abnormal cutoff score as greater than 10.

Insomnia was evaluated using the *Insomnia Severity Index (ISI)* ^28^ in adults and children. The total score is considered as pathological when it is higher than 10.

The severity of the disease was estimated using Narcolepsy Severity Scale (NSS),^29^ in children the item concerning the driving was not filled.

Health‐related quality of life (QoL) was assessed in adult patients with the *SF36 health survey*,^30^ including the physical, psychological, general well‐being as well as physical activity, pain, limitation, perceived heath, psychological health, vitality, and relations domains. QoL was evaluated in children and adolescents using a questionnaire named *Vécu et Santé Perçue* adapted for adolescents (11‐18 years) (VSP‐A)^31^ and for children (< 11 years) (VSP‐E).^32^ VSP is a self‐questionnaire exploring the following dimensions: psychological and physical well‐being, body image, vitality, friends, parents, teachers, medical staff, leisure, school performance, and a global QoL index (range: 0‐100). Lower scores correspond to a poorer quality of life.

### 2.3 Diagnosis procedure

The sleep and wake monitoring procedure included: (a) a complete clinical examination; (b) a sleep log of 15 days preceding the sleep laboratory evaluation; (c) a nighttime polysomnography; (d) followed by an MSLT with 4 to 5 standard nap opportunities every two hours which were terminated after 20 minutes if no sleep occurred, and after 15 minutes asleep if sleep occurred.^33^ The polysomnography included 3‐8 electroencephalograms, 2 electrooculograms, the surface electromyogram of the mentalis muscle and left and right anterior tibialis anterior muscles, nasal pressure recorded through cannulae, thoracic and abdominal belts, electrocardiogram. Sleep stages, arousals, motor, and respiratory events were scored visually according to standard pediatric and adult criteria by experienced sleep physicians.^33^ Mean sleep latency and number of SOREMPs were calculated on the MSLT, and the SOREMPs were normalized between patients with 4 or 5 tests.^33^

### 2.4 Criteria for narcolepsy with cataplexy

As CSF hypocretin levels were not done routinely for narcoleptic diagnosis in our sleep laboratories, we used the criteria for NC^1^ including (a) complaints of excessive daytime sleepiness occurring every day or almost every day for at least 3 months; (b) symptoms not better explained by other medical or psychiatric disorders; (c) absence of secondary narcolepsy; (d) the presence of clear‐cut cataplexy; (e) MSL lower than 8 minutes and two or more SOREMPs (including the MSLT and the previous night polysomnography). For this last item, we know that approximately 15% of adult patients with NC may have a normal or more borderline MSLT results (sleep latency of 8 minutes or longer or only one SOREMP (ICSD‐2 edition)). In a recent study, Pizza et al^34^ reported in a study evaluating the MSLT criteria in 357 children with narcolepsy type 1 that children with CSF hcrt‐1 deficiency were best recognized using a mean MSLT sleep latency ≤ 8.2 minutes or by at least 2 SOREMPs at the MSLT. HLA DQB1*0602 genotyping was performed in 28 adults and in 45 children.

### 2.5 Physical measures

Height and weight were measured, and body mass index (BMI) (weight/height^2^) was calculated. The BMI *z*‐score, which represents a measure of weight adjusted for height, sex, and age, relative to a smoothed reference distribution was computed.^35^ In adults, overweight was defined by a BMI greater than 25 kg/m^2^ and lower than 30, and obesity by a BMI greater than 30. In children, the World Health Organization (WHO) adolescence BMI‐for‐age curves at 19 years closely coincide with adult overweight (BMI 25) at + 1 SD and adult obesity (BMI 30) at + 2 SD. As a result, these SD classifications are extended down to 5 years.^36^ We used this definition in our study. Overweight and obesity were defined respectively when BMI was above + 1 SD and above + 2 SD for sex and age. Information concerning secondary sexual characteristics at symptoms onset were not known, and hence, the presence of precocious puberty could not be established. Early menarche was defined as menarche between age 9 and 11 years, using the 5th percentile of the French Health Behavior in school aged children distribution.^37^

### 2.6 Statistical analysis

Continuous measures were expressed as median and range. Pairwise comparisons between adults and children groups were performed using Wilcoxon tests for continuous measures because of the non‐normality of the distribution assessed by Shapiro‐Wilk test. Fisher's exact test was used for between‐group comparisons of dichotomous measures and chi‐square test for polytomous measures.

For continuous measures taken two by two, Spearman correlation was computed with the R function correlation test. As the items of QoL questionnaires were different between children and adults with NC, we restrained the comparison to the total QoL scores. Bivariate associations were performed to analyze the association between the QoL total score and the other measures. Owing to missing data, we had a different number of subjects for each couple of variables. We therefore computed the linear coefficient of correlation and the adjusted coefficient of determination after standardization of the two variables for each couple. In order to standardize a variable, we took the normal percentile of the rank of each value for a given couple. The coefficient of determination can then be interpreted as the percentage of variability of one variable “explained” by the other expressed in standard units. Package corrplot function was used to represent the coefficient of correlation. It was only possible in adult patients. There were too many missing data in children to perform these analyses.

Each continuous significant independent variable was then entered into the multivariate linear model. The significance level was set at 5%. Statistical calculations were performed using R (R CORE Team, version 2.15.2).^38^

## 3 RESULTS

### 3.1 General clinical characteristics

At time of NC diagnosis, the study group included 46 children (61% male) with a median age of 12 years old and 46 adults (61% male) with a median age of 28.5 years old, with no sex‐ratio difference. The BMI, BMI *z*‐score, and waist size were higher in adults than in children. Overweight was more prevalent in adults than in children; in contrast, obesity was more frequent in children than in adults. The age at menarche is similar between children and adults. The delay from disease onset to diagnosis seems to be equivalent between children and adults. Nine children and 4 adults had the H1N1 seasonal flu vaccination prior to disease onset. HLA DQB1*0602 positivity was more commonly found in children than in adults. There was no difference between groups for the CSF hypocretin values. Clinical characteristics of adults and children with NC are presented in Table 1.

**TABLE 1:** Clinical characteristics of adults and children with narcolepsy‐cataplexy

	Children	n	Adults	n	*P*
N (%)	46 (50)	46	46 (50)	46	—
Age, years	12 [5‐17]	46	28.5 [18‐71]	46	<.0001
Male, n (%)	28 (61)	46	28 (61)	46	1.00
Age at disease onset, years	10 [4‐15]	46	22 [8‐57]	46	<.0001
Age at sleepiness onset, years	10 [4‐15]	46	23 [8‐50]	41	<.0001
Age at cataplexy onset, years	12 [6‐15]	39	25 [15‐50]	37	<.0001
H1N1 vaccine prior to onset, n (%)	9 (36)	46	4 (14)	29	.76
HLA DQB1*0602, n (%)	44 (98)	45	24 (86)	28	<.0001
CSF hypocretin values (pg/mL)	5 [1‐90]	9	15 [1‐51]	8	.39
BMI, kg/m^2^	21.8 [14‐33.3]	46	25.5 [15.6‐36]	46	<.0001
Obesity, n (%)	25 (54)	46	8 (17)	46	.0004
Overweight, n (%)	6 (13)	46	20 (44)	46	.002
BMI *z*‐score	2.46 [−2.3‐10]	46	7.47 [0.2‐15.4]	46	<.0001
Waist size, cm	72 [58‐100]	15	86.5 [67‐122]	34	.007
Age at menarche, years	12 [10‐14]	11	12 [11‐15]	13	.39
Delay between disease onset and diagnosis, years	1 [0.3‐7]	46	3.5 [0‐50]	46	.21

Note

Measures are expressed as median and range [in brackets] or as N and percentage (in brackets). The *P* significance level was set at 5%.

Abbreviations: BMI, body mass index; CSF, cerebrospinal fluid; HLA, human leukocyte antigen genotyping.

### 3.2 Narcolepsy symptoms

In terms of timing, children had earlier narcolepsy symptoms (sleepiness, cataplexy, hypnagogic hallucinations, and sleep paralysis) than adults (Tables 1 and 2). The frequency and severity of excessive daytime sleepiness was similar between groups. However, sleep paralysis was more frequent in adults than in children. Hypnagogic hallucinations also tended to be more frequent in adults, but not significantly. In contrast, there were more frequent parasomnia, sleep talking, night eating, and sleep drunkenness (defined as being tired when waking up in the morning) in children than in adults. There was no difference between groups for the presence of dyssomnia (defined by poor quality of sleep with frequent awakenings), familial parasomnia, and severity of the disease. The total disease severity score was 26 (range from 2 to 54) in children vs 30.5 (range from 7 to 48) in adults. Narcolepsy related characteristics are presented in Table 2.

**TABLE 2:** Narcolepsy symptoms in adults and children with narcolepsy‐cataplexy

	Children	n	Adults	n	*P*
Sleepiness score at ESS/AESS (0‐24)	17 [12‐22]	37	18.5 [6‐24]	42	.08
Score > 10, n (%)	37 (100)	37	40/42 (95)	42	.50
Hypnagogic hallucinations, n (%)	21 (46)	46	30 (67)	45	.06
Age at hallucinations onset, years	10.5 [6‐15.1]	20	22 [8‐50]	25	<0.0001
Sleep paralysis, n (%)	9 (20)	45	24 (55)	44	.001
Age at sleep paralysis onset, years	13.5 [9‐16]	8	23 [8‐42]	21	.005
Dyssomnia, n (%)	17 (61)	28	21 (60)	35	1.00
Parasomnia, n (%)	41 (89)	46	17 (43)	40	<.0001
Sleep talking, n (%)	36 (80)	45	13 (34)	38	<.0001
Familial parasomnia, n (%)	14 (32)	44	9 (21)	43	.33
Sleep drunkenness, n (%)	29 (69)	42	11 (24)	45	<.0001
Night eating, n (%)	10 (29)	34	3 (7)	45	.01
Disease severity, score without item 7 (driving)	26 [2‐54]	33	29.5 [7‐45]	40	.11

Note

Data are expressed as median and range [in brackets] or as N and percentage (in brackets). Sleep drunkenness stands for severe sleep inertia. The significance level was chosen at 5%.

Abbreviations: AESS, Epworth sleepiness score adapted for children; ESS, Epworth sleepiness score.

The prevalence, frequency, and severity (partial vs total) of cataplexy were similar between groups (Table S1).

Although depressive feelings, insomnia, and fatigue symptoms did not differ between groups, the scores at the ADHD scale of Conners RS‐R were on average higher in children than in adults, with a higher number of children with a pathological score. Adults reported a lower quality of life than children. Results for mood, fatigue, insomnia, attention disorders, and quality of life are presented in Table 3.

**TABLE 3:** Mood, fatigue, insomnia, hyperactivity and quality of life in adults and children with narcolepsy‐cataplexy

	Children	n	Adults	n	*P*_Wilcoxon_/*P*_Fisher_
Depressive symptoms at the BDI/CDI, score	18 [3‐29]	33	10 [1‐31]	38	.14
Score ≥ 16, n (%)	8 (24)	33	12 (32)	38	.60
Chalder's fatigue scale, score	8 [3‐14]	32	9 [0‐14]	38	.66
Score > 10, n (%)	8 (25)	32	14 (37)	38	.31
Conners RS‐R, score	50 [35‐88]	29	26 [6‐52]	37	<.0001
Score > 65, n (%)	6 (21)	29	0 (0)	37	<.001
Score > 75, n (%)	2 (7)	29	0 (0)	37	.20
Insomnia severity index, score	13 [2‐22]	33	15 [7‐26]	37	.52
Score > 10, n (%)	28 (85)	33	28 (76)	37	.38
Quality of life, total score	59 [34‐87]	27	43 [31‐58]	39	<.0001

Note

Data are expressed as median [range] or number (frequency). The significance level was set at 5%.

Abbreviations: BDI, Beck Depression Inventory; CDI, Children's Depression Inventory; Conners RS‐R, Conners’ Rating Scale‐Revised.

### 3.3 Sleep measures

On nighttime polysomnography, the total sleep time was longer in children than in adults (Table 4). No difference was found between groups concerning sleep and REM sleep onset latencies, sleep efficiency. Although there was no difference for the percentage of REM stage between groups, there was a higher percentage of stage 1 sleep and wake time after sleep onset, a lower percentage of stage 2 sleep and a trend for higher percentage of N3 stage in children than in adults. Children had lower obstructive apnea/hypopnea index (OAHI) than adults and a lower proportion of children had OAHI ≥ 5. The mean and minimal transcutaneous oxygen saturation were lower in adults than in children. Higher BMI z‐score were correlated with higher OAHI (*r* = .32, *P* = .002).

**TABLE 4:** Nighttime and daytime sleep measures upon diagnosis evaluation (without treatment) in adults and children with narcolepsy‐cataplexy

	Children	n	Adults	n	*P*
TST, min	467 [270‐640]	45	437 [257‐534]	46	.03
Sleep efficiency, %	83.8 [52.5‐95.2]	45	86 [57‐97]	45	.55
Wake after sleep onset, min	91 [18‐260]	45	47 [2‐209]	45	.02
Latency to					
Sleep onset, min	4 [0‐78]	45	4.5 [0‐228]	46	.63
REM sleep onset, min	19.8 [0‐394]	45	23.4 [0‐367]	46	.68
Stage N1, %	14.3 [0.1‐35.8]	45	8.1 [0.9‐24]	46	.03
Stage N2, %	43.3 [16.1‐56.2]	45	46 [30.3‐45.5]	46	.01
Stage N3, %	22.1 [9.9‐48.7]	45	20 [5.6‐52.9]	46	.09
Stage REM, %	22.5[4‐32.6]	45	20.7 [6.3‐38.6]	46	0.78
OAHI, n/h	0.60 [0‐22.4]	45	2.3 [0‐45.1]	45	.007
OAHI ≥ 5/, n/h TST	3 (7)	45	14 (31)	45	.006
Mean oxygen saturation, %	97 [94.2‐99.3]	45	96 [92‐100]	39	.01
Minimal oxygen saturation, %	93.8 [69.3‐98]	42	91 [81‐95]	42	<.0001
*Multiple sleep latency tests,* n (%)	46 (100)	46	46 (100)	46	1.00
N 5 tests, n (%)	15 (33)	46	46 (100)	46	<.0001
Mean sleep latency, min	3.2 [0.5‐10.2]	46	3.9 [0.6‐12.6]	46	.05
Sleep onset REM periods, n	4 [1‐5]	46	4 [2‐5]	46	.57
SOREM/N tests (%)	100 [25‐100]	46	80 [40‐100]	46	.002

Note

Data are expressed as median [range] or number (frequency). The significance level was chosen at 5%.

Abbreviations: OAHI, Obstructive Apnea‐hypopnea index; REM, rapid eye movement sleep; Stage N3, NREM3 (Slow‐Wave Sleep); TST, Total Sleep Time.

As for MSLT, the MSL tended to be lower in children than in adults. The number of SOREMPs was similar across groups; however, the number of SOREM/MSLT tests was higher in children than in adults. Two adult patients had sleep latencies > 8 minutes (range 11.8 to 12.6 minutes) on MSLT, and all these patients had clear‐cut cataplexy and 4 SOREM. For children, respectively, 4 children had sleep latencies ≥ 8 minutes (8‐10.2 minutes), and two had sleep latencies > 8.2 minutes (9.4‐10.2 minutes), all with at least 2 SOREM. Two children had less than 2 SOREM but all with sleep latency less than 8 minutes.

### 3.4 Determinants of quality of life

**FIGURE 1:**
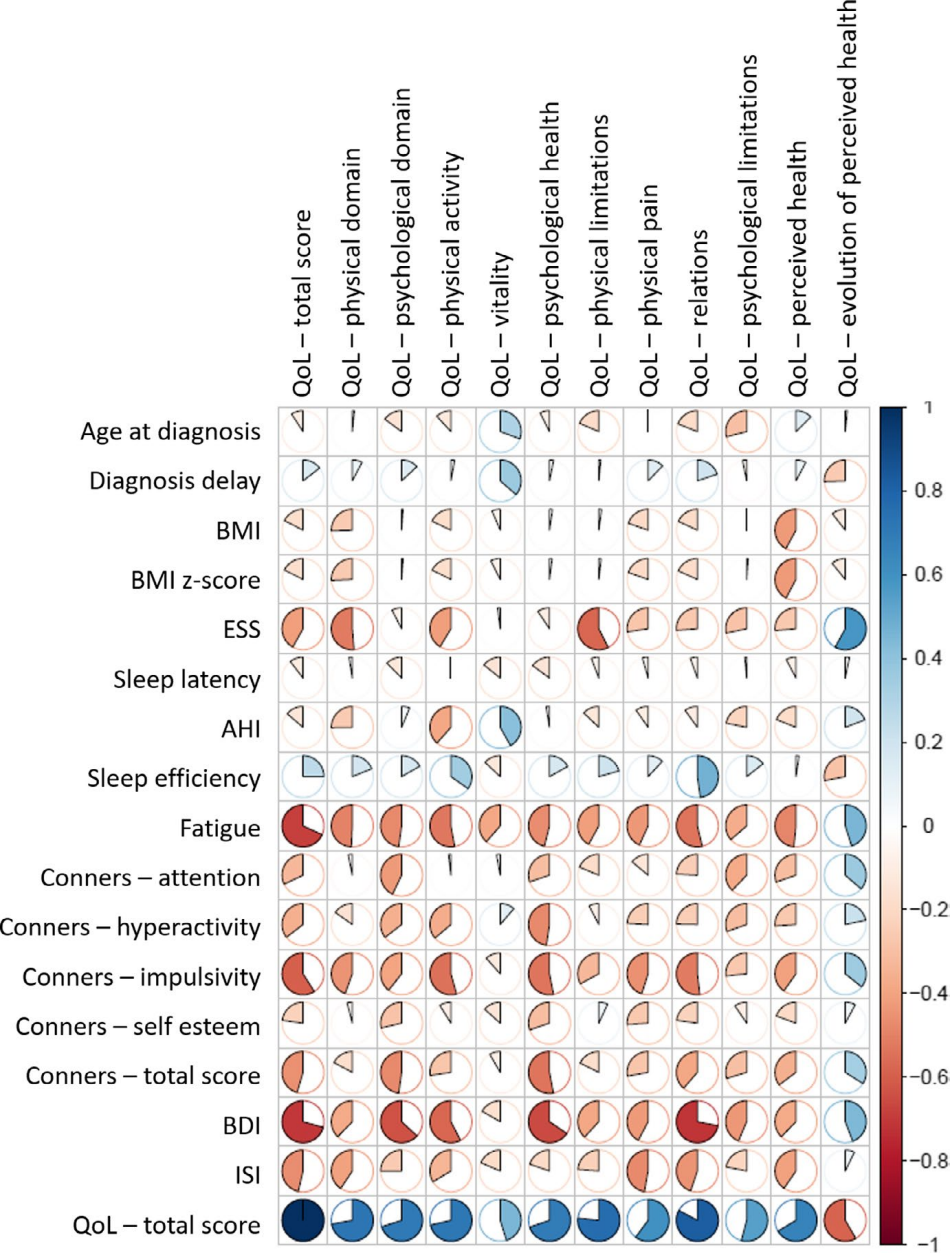
Correlation plot for adults. Bivariate associations between the quality of life dimensions and the continuous covariates in adult narcolepsy‐cataplexy. Positive and negative associations in blue and red, respectively. AHI, apnea‐hypopnea index; BDI, Beck depression inventory; BMI, body mass index; ESS, Epworth sleepiness scale; QoL, quality of life; ISI, Insomnia severity index

In adult patients, the bivariate association between the QoL dimensions and the continuous covariates is provided in Supplemental Table 2 and in Figure 1. Depression and fatigue were the factors that most affected the quality of life (46% for both), followed by behavioral problems (impulsivity) (35%), insomnia (23%), daytime sleepiness (20%), hyperactivity (12%), and self‐confidence (9%). These factors mostly impacted psychological well‐being and social life.

**FIGURE 2:**
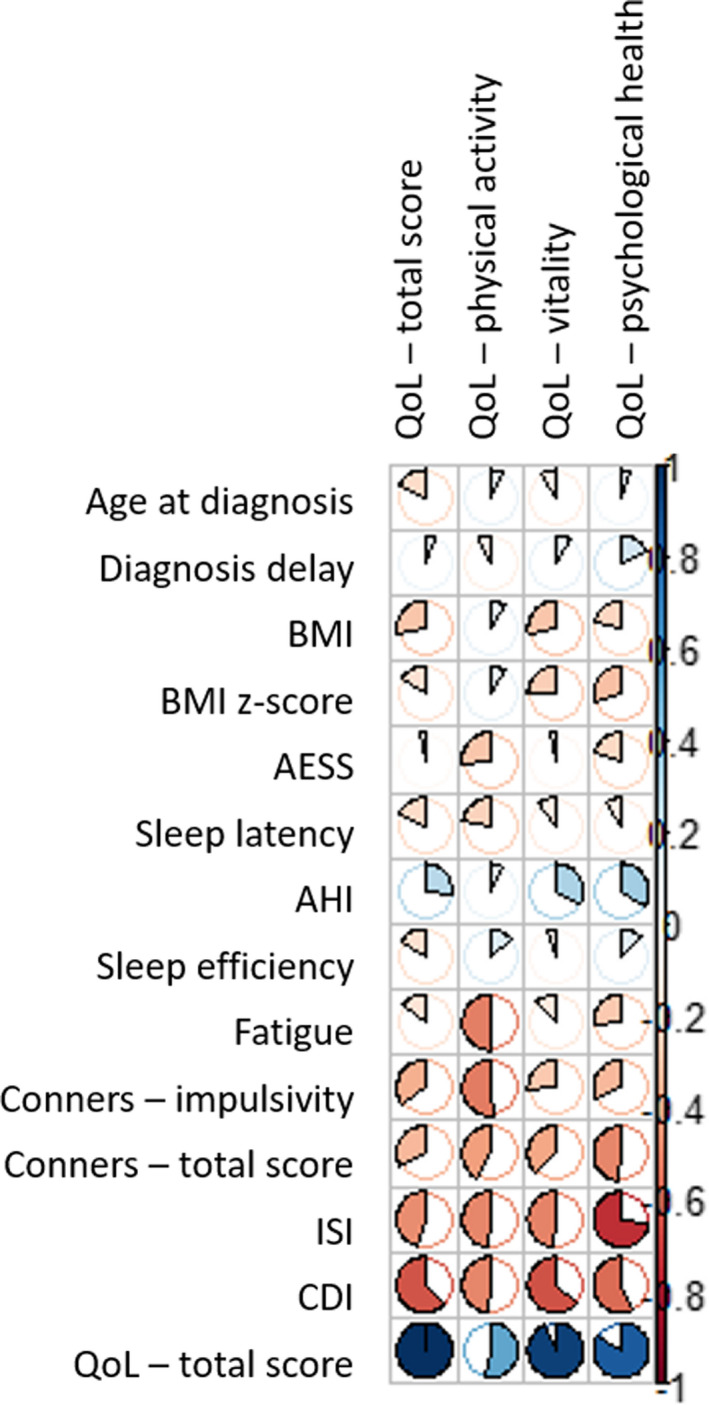
Correlation plot for children. Bivariate associations between the quality of life dimensions and the continuous covariates in children narcolepsy‐cataplexy. Positive and negative associations in blue and red, respectively. AHI, apnea‐hypopnea index; BMI, body mass index; CDI, children's depression inventory; ESS, Epworth sleepiness scale; ISI, Insomnia severity index; QoL, quality of life

In children (Table S3 and Figure 2), depression was the factor that most affected the quality of life (44%), followed by insomnia (30%) and ADHD symptoms (17%). The factors mostly impacted psychological health and physical activity. However, there were few data for children.

In the multivariate regression model in children, when the CDI score was entered into the linear model adjusted for gender and age, no other continuous independent variable could significantly increase the likelihood of the model. The retained model with gender, age, and CDI score as independent variables “explained” 49% of the variability of the QoL score (N = 27).

In adults, the retained model with gender, age and Beck and fatigue scores as independent variables “explained” 56% of the variability of the QoL score (N = 36). In adults, QoL was mostly impacted by depression but also by fatigue and ESS scores. Similar results were found between Beck and Epworth scores, and these independent variables explained 52% of the variability of the QoL score (N = 38). There was a positive correlation between fatigue and Epworth scores in adults (***r*** *= *.40, *P* = .01).

## 4 DISCUSSION

This study indicates that children and adults suffering from NC present different clinical characteristics and sleep measures.

Obesity was the most frequent comorbid problem here in children and adults with NC, as 54% of children were obese compared to 17% of adults. This frequency is higher in childhood NC than in the general population, as 17% of children were overweight and 4% were obese in France.^39^ Several previous studies highlighted this high frequency of obesity in childhood NC.^14^, ^15^ Indeed, the association of NT1 with obesity and precocious puberty in children (not measured here) could reflect broad and severe hypothalamic abnormalities.^14^, ^15^ In contrast, the prevalence of overweight and obesity in adults with NC was somehow close to that of the general population, as 49% of adults were overweight and 17% were obese in France.^39^ Different mechanisms could be implicated in the high obesity prevalence observed in childhood narcolepsy. Experimental studies suggest that the hypocretin system could play a role in the regulation of metabolism.^40^ However, in the few patients reported in our study, no differences were found in CSF hypocretin‐1 levels between children and adults. We recently reported an impairment in histamine neurotransmission, another brain wake‐promoting system, in children with NT1,^41^ which was not found in adults.^42^ Histaminergic neurons could be also involved as studies in animal models have suggested that lack of either hypocretin or histamine could lead to a mild obesity.^43^, ^44^ Moreover, various evidences from animal models and adult patients support the idea that both decreased metabolism and subtle changes in eating behavior (rather than in calorie intake) are responsible for the positive energy balance leading to obesity in narcolepsy. However, this difference in metabolism was not consistent across adult studies.^17^, ^45^ In children with NT1, Wang et al^46^ recently documented a weight gain and decrease in basal metabolic rate (BMR) in the early stage of the disease (≤12 months) compared to control children. In the present study, we found more night eating in children compared to adults with NC.

As a consequence of sleep maturation, children need more sleep, particularly nonrapid eye movement (NREM) sleep. The presence of higher frequency of sleep drunkenness in narcoleptic children could be also explained by this increase need of sleep or by their lower sleep quality as showed by more light N1 sleep and wake time after sleep onset compared to narcoleptic adults. Moreover, NREM parasomnia was more frequent in children than in adults with NC which is consistent with what is observed in the general pediatric population.^47^ However, RBD as a REM parasomnia has also been reported in up to 60% of adult patients, but also in children with narcolepsy.^48^ It can also not be excluded that children in the present study had REM sleep parasomnia. REM sleep‐associated abnormal behaviors such as sleep paralysis occurred more frequently in adults than in children with narcolepsy, as shown previously between adolescents and young children with NC.^12^

Although less obese, adult patients had more OSA than children. It has been shown that children and adolescents, with or without obesity, have less upper airway collapsibility compared to adult population.^49^, ^50^, ^51^, ^52^ This could explain that OSA is seen more commonly among older people for the same degree of obesity. In line with results from the general population, Nevsimalova et al^18^ found that 53% of older narcoleptic patients (over 60 years of age) presented OSA while no young patients (under 20 years) did.

Furthermore, ADHD symptoms were found in about a quarter of children while none were reported in adults. Deficiency in alertness and sleep disturbances have been hypothesized to contribute to ADHD in previous studies.^53^, ^54^ Lecendreux et al^55^ found that pediatric narcoleptic patients had approximately twofold higher ADHD symptoms, compared to controls. Filardi et al^56^ found that as well adult narcoleptic patients had higher severity of ADHD inattentive scores than controls. However, if ADHD persist across life in narcoleptic patients, ADHD symptoms normally tend to decrease with cerebral maturation especially impulsivity and hyperactivity.

Adult patients herein had lower quality of life than narcoleptic children. As previously reported, narcoleptic patients experience difficulties in social relationships, whether in childhood or adulthood.^57^, ^58^, ^59^, ^60^ In both children and adults, depression was the factor that most affected the quality of life in patients.^57^, ^59^ The lower quality of life in adulthood NC could be attributable to the fact that in adult populations, work and marital situation play a more important role than in the case of adolescents who are attending school. In the study by White et al,^59^ the authors found that even if no difference was observed in occupational category and professional status, patients expressed significantly less satisfaction about their work and a less favorable professional career than controls did.

The present study has some limitations. We analyzed only the data of 46 adult patients. Indeed, in the NARCOBANK database, a large number of adult patients were already treated and we kept only for this study de novo patients presenting narcolepsy with cataplexy without treatment. Although the same protocols were followed, the questionnaires were different in children and adults. Some questionnaires were not filled by the patients, and there were some missing data. For MSLT, the whole group of adults experienced 5 naps whereas 31 children underwent only 4 naps. Not all patients had a lumbar puncture to confirm the decrease in hypocretin levels. Since this was a retrospective study, it would be interesting to conduct a prospective study in these children to follow their outcome during adult life.

In conclusion, compared to NC adults, children with NC presented with a higher prevalence of obesity, night eating, parasomnia, sleep talking, and ADHD. Conversely, adult patients showed more sleep paralysis, OSA, and a lower quality of life. Such differences in clinical characteristics and complications according to age need to be considered in order to ensure a prompt diagnosis and better management to prevent such complications.

## CONFLICT OF INTEREST

The authors declare no conflict of interest.

## Supporting information

Supplementary Material

Supplementary Material

## References

[1] American Academy of Sleep Medicine . International classification of sleep disorders, 2nd ed Diagnostic and Coding Manual. Westchester, IL: American Academy of Sleep Medicine 2005.

[2] Ohayon MM , Priest RG , Zulley J , Smirne S , Paiva T . Prevalence of narcolepsy symptomatology and diagnosis in the European general population. Neurology. 2002;58:1826‐1833. 12084885 10.1212/wnl.58.12.1826

[3] Silber MH , Krahn LE , Olson EJ , Pankratz VS . The epidemiology of narcolepsy in Olmsted County, Minnesota: a population‐based study. Sleep. 2002;25:197‐202. 11902429 10.1093/sleep/25.2.197

[4] Dauvilliers Y , Arnulf I , Lecendreux M , et al. Increased risk of narcolepsy in children and adults after pandemic H1N1 vaccination in France. Brain. 2013;136:2486‐2496. 23884811 10.1093/brain/awt187

[5] Han F , Lin L , Warby SC , et al. Narcolepsy onset is seasonal and increased following the 2009 H1N1 pandemic in china. Ann Neurol. 2011;70:410‐417. 21866560 10.1002/ana.22587

[6] Peyron C , Faraco J , Rogers W , et al. A mutation in a case of early onset narcolepsy and a generalized absence of hypocretin peptides in human narcoleptic brains. Nat Med. 2000;6:991‐997. 10973318 10.1038/79690

[7] Cvetkovic‐Lopes V , Bayer L , Dorsaz S , et al. Elevated Tribbles homolog 2‐specific antibody levels in narcolepsy patients. J Clin Invest. 2010;120:713‐719. 20160349 10.1172/JCI41366 PMC2827962

[8] Aran A , Lin L , Nevsimalova S , et al. Elevated anti‐streptococcal antibodies in patients with recent narcolepsy onset. Sleep. 2009;32:979‐983. 19725248 10.1093/sleep/32.8.979 PMC2717204

[9] Mignot E , Lin L , Rogers W , et al. Complex HLA‐DR and ‐DQ interactions confer risk of narcolepsy‐cataplexy in three ethnic groups. Am J Hum Genet. 2002;68:686‐699. 10.1086/318799 PMC1274481 11179016

[10] Nevsimalova S , Buskova J , Kemlink D , Sonka K , Skibova J . Does age at the onset of narcolepsy influence the course and severity of the disease? Sleep Med. 2009;10:967‐972. 19423388 10.1016/j.sleep.2009.01.010

[11] Morrish E , King MA , Smith IE , Shneerson JM . Factors associated with a delay in the diagnosis of narcolepsy. Sleep Med. 2004;5:37‐41. 14725825 10.1016/j.sleep.2003.06.002

[12] Aran A , Einen M , Lin L , Plazzi G , Nishino S , Mignot E . Clinical and therapeutic aspects of childhood narcolepsy‐cataplexy: a retrospective study of 51 children. Sleep. 2010;33:1457‐1464. 21102987 10.1093/sleep/33.11.1457 PMC2954695

[13] Dauvilliers Y , Gosselin A , Paquet J , Touchon J , Billiard M , Montplaisir J . Effect of age on MSLT results in patients with narcolepsy‐cataplexy. Neurology. 2004;62:46‐50. 14718696 10.1212/01.wnl.0000101725.34089.1e

[14] Inocente CO , Lavault S , Lecendreux M , et al. Impact of obesity in children with narcolepsy. CNS Neurosci Ther. 2013;19:521‐528. 23574649 10.1111/cns.12105 PMC6493658

[15] Poli F , Pizza F , Mignot E , et al. High prevalence of precocious puberty and obesity in childhood narcolepsy with cataplexy. Sleep. 2013;36:175‐181. 23372264 10.5665/sleep.2366 PMC3543059

[16] Schuld A , Hebebrand J , Geller F , Pollmacher T . Increased body‐mass index in patients with narcolepsy. Lancet. 2000;355:1274‐1275. 10.1016/S0140-6736(05)74704-8 10770327

[17] Dahmen N , Tonn P , Messroghli L , Ghezel‐ahmadi D , Engel A . Basal metabolic rate in narcoleptic patients. Sleep. 2009;32:962‐964. 19639760 PMC2706907

[18] Nevsimalova S , Pisko J , Buskova J , et al. Narcolepsy: clinical differences and association with other sleep disorders in different age groups. J Neurol. 2013;260:767‐775. 23070467 10.1007/s00415-012-6702-4

[19] Johns MW . A new method for measuring daytime sleepiness: the Epworth sleepiness scale. Sleep. 1991;14:540‐545. 1798888 10.1093/sleep/14.6.540

[20] Snow A , Gozal E , Malhotra A , et al. Severe hypersomnolence after pituitary/hypothalamic surgery in adolescents: clinical characteristics and potential mechanisms. Pediatrics. 2002;110:e74. 12456941 10.1542/peds.110.6.e74

[21] Murali H , Kotagal S . Off‐label treatment of severe childhood narcolepsy‐cataplexy with sodium oxybate. Sleep. 2006;29:1025‐1029. 16944670 10.1093/sleep/29.8.1025

[22] Dauvilliers Y , Montplaisir J , Molinari N , et al. Age at onset of narcolepsy in two large populations of patients in France and Quebec. Neurology. 2001;57:2029‐2033. 11739821 10.1212/wnl.57.11.2029

[23] Van Beek Y , Hessen DJ , Hutteman R , Verhulp EE , Van Leuven M . Age and gender differences in depression across adolescence: real or “bias”? J Child Psychol Psychiatry Allied Discip. 2012;53:973‐985. 10.1111/j.1469-7610.2012.02553.x 22512614

[24] Kovacs M . The Children’s Depression, Inventory (CDI). Psychopharmacol Bull. 1985;21:995‐998. 4089116

[25] Christiansen H , Kis B , Hirsch O , et al. German validation of the Conners Adult ADHD Rating Scales‐self‐report (CAARS‐S) I: factor structure and normative data. Eur Psychiatry. 2011;26:100‐107. 20619613 10.1016/j.eurpsy.2009.12.024

[26] Goyette CH , Conners CK , Ulrich RF . Normative data on Revised Conners Parent and Teacher Rating Scales. J Abnorm Child Psychol. 1978;6:221‐236. 670589 10.1007/BF00919127

[27] Chalder T , Berelowitz G , Pawlikowska T , et al. Development of a fatigue scale. J Psychosom Res. 1993;37:147‐153. 8463991 10.1016/0022-3999(93)90081-p

[28] Bastien CH , Vallières A , Morin CM . Validation of the insomnia severity index as an outcome measure for insomnia research. Sleep Med. 2001;2:297‐307. 11438246 10.1016/s1389-9457(00)00065-4

[29] Dauvilliers Y , Barateau L , Lopez R , et al. Narcolepsy Severity Scale: a reliable tool assessing symptom severity and consequences. Sleep. 2020;43(6):zsaa009 10.1093/sleep/zsaa009 31993661

[30] Ware JE , Sherbourne CD . The MOS 36‐item short‐form health survey (SF‐36) I. conceptual framework and item selection. Med Care. 1992;30:473‐483. 1593914

[31] Simeoni MC , Auquier P , Antoniotti S , Sapin C , San Marco JL . Validation of a French health‐related quality of life instrument for adolescents: the VSP‐A. Qual Life Res. 2000;9:393‐403. 11131932 10.1023/a:1008957104322

[32] Gras D . Santé et qualité de vie des frères et sœurs d’enfants atteints de maladies chroniques. Nantes: Medical Thesis, Nantes Medical University; 2009.

[33] Iber C , Ancoli‐Israel S , Chesson A , Quan SF . The AASM manual for the scoring of sleep and associated events: rules, terminology and technical specification. J Clin Sleep Med. 2007;3:752. 18198811 PMC2556904

[34] Pizza F , Barateau L , Jaussent I , et al. Validation of multiple sleep latency test for the diagnosis of pediatric narcolepsy type 1. Neurology. 2019;93:e1034‐e1044. 31405906 10.1212/WNL.0000000000008094

[35] Cole TJ , Bellizzi MC , Flegal KM , Dietz WH . Establishing a standard definition for child overweight and obesity worldwide: international survey. BMJ. 2000;320:1240‐1243. 10797032 10.1136/bmj.320.7244.1240 PMC27365

[36] De Onis M , Lobstein T . Defining obesity risk status in the general childhood population: which cut‐offs should we use? Int J Pediatr Obes. 2010;5:458‐460. 20233144 10.3109/17477161003615583

[37] Gaudineau A , Ehlinger V , Vayssiere C , Jouret B , Arnaud C , Godeau E . Factors associated with early menarche: results from the French health behaviour in school‐aged children (HBSC) study. BMC Public Health. 2010;10:1‐7. 20353570 10.1186/1471-2458-10-175 PMC2853511

[38] R Development CORE TEAM . 2009: R: a language and environment for statistical computing. Vienna, Austria 2012: Internet: http://www.R‐project.org

[39] Verdot C , Torres M , Salanave B , Deschamps V . Corpulence des enfants et des adultes en France métropolitaine en 2015. Résultats de l’étude Esteban et évolution depuis 2006. Bull Epidémiol Hebd. 2015;2017(13):234‐241.

[40] Skrzypski M , T. Le T , Kaczmarek P , et al. Orexin A stimulates glucose uptake, lipid accumulation and adiponectin secretion from 3T3‐L1 adipocytes and isolated primary rat adipocytes. Diabetologia. 2011;54:1841‐1852. 21505958 10.1007/s00125-011-2152-2

[41] Franco P , Dauvilliers Y , Inocente CO , et al. Impaired histaminergic neurotransmission in children with narcolepsy type 1. CNS Neurosci Ther. 2019;25:386‐395. 30225986 10.1111/cns.13057 PMC6488909

[42] Dauvilliers Y , Delallée N , Jaussent I , et al. Normal cerebrospinal fluid histamine and tele‐Methylhistamine levels in hypersomnia conditions. Sleep. 2012;35:1359‐1366. 23024434 10.5665/sleep.2114 PMC3443762

[43] Anaclet C , Parmentier R , Ouk K , et al. Orexin/hypocretin and histamine: distinct roles in the control of wakefulness demonstrated using knock‐out mouse models. J Neurosci. 2009;29:14423‐14438. 19923277 10.1523/JNEUROSCI.2604-09.2009 PMC2802289

[44] Parmentier R , Ohtsu H , Djebbara‐Hannas Z , Valatx J‐L , Watanabe T , Lin J‐S . Anatomical, physiological, and pharmacological characteristics of histidine decarboxylase knock‐out mice: evidence for the role of brain histamine in behavioral and sleep‐wake control. J Neurosci. 2002;22:7695‐7711. 12196593 10.1523/JNEUROSCI.22-17-07695.2002 PMC6757981

[45] Fronczek R , Overeem S , Reijntjes R , Lammers GJ , Van Dijk JG , Pijl H . Increased heart rate variability but normal resting metabolic rate in hypocretin/orexin‐deficient human narcolepsy. J Clin Sleep Med. 2008;4:248‐254. 18595438 PMC2546458

[46] Wang Z , Wu H , Stone WS , et al. Body weight and basal metabolic rate in childhood narcolepsy: a longitudinal study. Sleep Med. 2016;25:139‐144. 27823707 10.1016/j.sleep.2016.06.019

[47] Laberge L , Tremblay RE , Vitaro F , Montplaisir J , PhD C . Development of parasomnias from childhood to early adolescence. Pediatrics. 2004;106:67‐74. 10.1542/peds.106.1.67 10878151

[48] Nevsimalova S , Prihodova I , Kemlink D , Lin L , Mignot E . REM behavior disorder (RBD) can be one of the first symptoms of childhood narcolepsy. Sleep Med. 2007;8:784‐786. 17569582 10.1016/j.sleep.2006.11.018

[49] Isono S , Saeki N , Tanaka A , Nishino T . Collapsibility of passive pharynx in patients with acromegaly. Am J Respir Crit Care Med. 1999;160:64‐68. 10390381 10.1164/ajrccm.160.1.9806054

[50] Isono S , Tanaka A , Ishikawa T , Nishino T . Developmental changes in collapsibility of the passive pharynx during infancy. Am J Respir Crit Care Med. 2000;162:832‐836. 10988091 10.1164/ajrccm.162.3.9911089

[51] Marcus CL , McColley SA , Carroll JL , Loughlin GM , Smith PL , Schwartz AR . Upper airway collapsibility in children with obstructive sleep apnea syndrome. J Appl Physiol. 1994;77:918‐924. 8002548 10.1152/jappl.1994.77.2.918

[52] Marcus CL , Keenan BT , Huang J , et al. The obstructive sleep apnoea syndrome in adolescents. Thorax. 2017;72:720‐728. 27503232 10.1136/thoraxjnl-2016-208660 PMC9277419

[53] Cortese S , Konofal E , Lecendreux M . Alertness and feeding behaviors in ADHD: does the hypocretin/orexin system play a role? Med Hypotheses. 2008;71:770‐775. 18678446 10.1016/j.mehy.2008.06.017

[54] Lecendreux M , Konofal E , Bouvard M , Falissard B , Mouren‐Siméoni MC . Sleep and alertness in children with ADHD. J Child Psychol Psychiatry Allied Discip. 2000;41:803‐812. 11039692

[55] Lecendreux M , Lavault S , Lopez R , et al. Attention‐Deficit/Hyperactivity Disorder (ADHD) symptoms in pediatric narcolepsy: a cross‐sectional study. Sleep. 2015;38:1285‐1295. 26118560 10.5665/sleep.4910 PMC4507734

[56] Filardi M , Pizza F , Tonetti L , Antelmi E , Natale V , Plazzi G . Attention impairments and ADHD symptoms in adult narcoleptic patients with and without hypocretin deficiency. PLoS One. 2017;12:e0182085. 28763482 10.1371/journal.pone.0182085 PMC5538711

[57] Inocente CO , Gustin M‐P , Lavault S , et al. Quality of life in children with narcolepsy. CNS Neurosci Ther. 2014;20:763‐771. 24922610 10.1111/cns.12291 PMC6493048

[58] Ingravallo F , Gnucci V , Pizza F , et al. The burden of narcolepsy with cataplexy: How disease history and clinical features influence socio‐economic outcomes. Sleep Med. 2012;13:1293‐1300. 23026503 10.1016/j.sleep.2012.08.002

[59] White M , Charbotel B , Fort E , et al. Academic and professional paths of narcoleptic patients: the NARCOWORK study. Sleep Med. 2019;65:96‐104. 31739232 10.1016/j.sleep.2019.07.020

[60] Dodel R , Peter H , Spottke A , et al. Health‐related quality of life in Portuguese patients with narcolepsy. Sleep Med. 2007;8:733‐741. 17512797 10.1016/j.sleep.2006.10.010

