# Supplementary material for: Narcolepsy with cataplexy: Does age at diagnosis change the clinical picture?
Source: CNS Neurosci Ther. 2020 Aug 6;26(10):1092–102. doi: 10.1111/cns.13438 (PMC7539846; doi:10.1111/cns.13438)
Supplement: Supplementary file 1 — Supplementary Material [file CNS-26-1092-s001.docx]

Methods

Patients

All children presenting with idiopathic narcolepsy with or without

cataplexy (age < 18 years old) seen in the four national reference

centers for narcolepsy between 2008 and 2011 were included in

the study (research program: NARCOBANK) [1]. Twenty-three

Supplementary material 1

**Table S1: Cataplexy characteristics in adults and children with narcolepsy-cataplexy**

|  | **Children** | **n** | **Adults** | **n** | ***p*** |
| --- | --- | --- | --- | --- | --- |
| Cataplexy, n (%) | 46 (100) | 46 | 46 (100) | 46 | 1.00 |
| **Partial cataplexy, n (%)** | 42 (100) | 42 | 39 (100) | 39 | 1.00 |
| **Partial cataplexy frequency** |  | 39 |  | 36 | 0.16 |
| < 1/year, n (%) | 1 (3) |  | 0 (0) |  |  |
| >1/year, n (%) | 0 (0) |  | 3 (8) |  |  |
| >1/month, n (%) | 4 (10) |  | 8 (22) |  |  |
| > 1/week, n (%) | 13 (33) |  | 10 (28) |  |  |
| >1/day, n (%) | 21 (54) |  | 15 (42) |  |  |
| **Total cataplexy, n (%)** | 27 (64) | 42 | 23 (59) | 39 | 0.65 |
| **Total cataplexy frequency** |  | 34 |  | 22 | 0.13 |
| < 1/year, n (%) | 9 (26) |  | 3 (14) |  |  |
| >1/year, n (%) | 2 (7) |  | 5 (23) |  |  |
| >1/month, n (%) | 10 (29) |  | 5 (23) |  |  |
| > 1/week, n (%) | 9 (26) |  | 3 (14) |  |  |
| >1/day, n (%) | 4 (12) |  | 6 (26) |  |  |

Data are expressed as N and percentage (in brackets). The significance level was set at 5%.
