# Supplementary material for: Narcolepsy with cataplexy: Does age at diagnosis change the clinical picture?
Source: CNS Neurosci Ther. 2020 Aug 6;26(10):1092–102. doi: 10.1111/cns.13438 (PMC7539846; doi:10.1111/cns.13438)
Supplement: Supplementary file 2 — Supplementary Material [file CNS-26-1092-s002.docx]

Methods

Patients

All children presenting with idiopathic narcolepsy with or without

cataplexy (age < 18 years old) seen in the four national reference

centers for narcolepsy between 2008 and 2011 were included in

the study (research program: NARCOBANK) [1]. Twenty-three

Supplemental material 2

Tables 2a. Pearson correlation matrix for adults. Each coefficient represents the linear correlation (**r**) between two variables.

| **Pearson correlation coefficient** | **Age at diagnosis** | **Diagnostic delay** | **BMI** | **BMI z-score** | **AESS** | **Sleep latency** | **AHI** | **Sleep efficiency** | **Fatigue score** | **Attention Conners** | **Hyperactivity Conners** | **Impulsivity Conners** | **Self-Confidence Conners** | **Total Conners** | **BECK** | **ISI** | **General well being** | **Physical well being** | **Psychological well being** | **Physical activity** | **Vitality** | **psychological health** | **Physical limitation** | **Physical pain** | **Relations** | **Psychological limitation** | **Perceived health** | **Evolution of Perceived health** |
| --- | --- | --- | --- | --- | --- | --- | --- | --- | --- | --- | --- | --- | --- | --- | --- | --- | --- | --- | --- | --- | --- | --- | --- | --- | --- | --- | --- | --- |
| **Age at diagnosis** |  | 0,65 | 0,21 | 0,21 | 0,06 | -0,16 | 0,56 | -0,42 | -0,10 | 0,06 | -0,09 | -0,06 | -0,30 | -0,07 | 0,08 | 0,10 | 0,04 | 0,12 | -0,06 | -0,02 | 0,33 | 0,14 | -0,17 | 0,15 | -0,08 | -0,33 | 0,26 | 0,00 |
| **Diagnostic delay** | 0,65 |  | 0,04 | 0,04 | 0,15 | -0,07 | 0,54 | -0,30 | -0,24 | 0,17 | 0,04 | -0,13 | -0,20 | -0,02 | -0,12 | -0,06 | 0,24 | 0,13 | 0,21 | 0,08 | 0,31 | 0,23 | 0,07 | 0,18 | 0,27 | -0,06 | 0,15 | -0,23 |
| **BMI** | 0,21 | 0,04 |  | 1,00 | -0,12 | -0,08 | 0,29 | -0,04 | -0,18 | -0,16 | 0,09 | 0,12 | 0,11 | 0,01 | 0,09 | 0,23 | -0,09 | -0,10 | -0,03 | -0,15 | 0,09 | -0,01 | -0,04 | -0,08 | -0,17 | -0,07 | -0,05 | -0,13 |
| **BMI z-score** | 0,21 | 0,04 | 1,00 |  | -0,12 | -0,08 | 0,29 | -0,04 | -0,18 | -0,16 | 0,09 | 0,12 | 0,11 | 0,01 | 0,09 | 0,24 | -0,09 | -0,10 | -0,03 | -0,15 | 0,08 | -0,02 | -0,04 | -0,08 | -0,17 | -0,06 | -0,05 | -0,13 |
| **AESS** | 0,06 | 0,15 | -0,12 | -0,12 |  | -0,05 | 0,18 | -0,29 | 0,40 | 0,21 | 0,09 | 0,25 | 0,00 | 0,20 | 0,19 | 0,14 | -0,45 | -0,53 | -0,12 | -0,43 | -0,07 | -0,18 | -0,44 | -0,35 | -0,28 | -0,22 | -0,30 | 0,49 |
| **Sleep latency** | -0,16 | -0,07 | -0,08 | -0,08 | -0,05 |  | -0,13 | 0,05 | 0,01 | 0,10 | -0,07 | -0,23 | -0,17 | -0,13 | -0,02 | -0,23 | 0,01 | -0,14 | 0,15 | 0,00 | -0,11 | 0,00 | 0,10 | -0,17 | 0,23 | 0,14 | -0,18 | 0,10 |
| **AHI** | 0,56 | 0,54 | 0,29 | 0,29 | 0,18 | -0,13 |  | -0,36 | -0,04 | 0,07 | 0,17 | 0,10 | -0,07 | 0,09 | 0,26 | 0,19 | -0,08 | -0,14 | 0,03 | -0,29 | 0,33 | 0,06 | -0,16 | 0,04 | -0,08 | -0,27 | 0,02 | 0,14 |
| **Sleep efficiency** | -0,42 | -0,30 | -0,04 | -0,04 | -0,29 | 0,05 | -0,36 |  | -0,05 | -0,29 | 0,06 | -0,11 | -0,25 | -0,19 | -0,35 | -0,16 | 0,22 | 0,18 | 0,13 | 0,26 | -0,01 | 0,08 | 0,17 | 0,09 | 0,37 | 0,12 | 0,05 | -0,17 |
| **Fatigue score** | -0,10 | -0,24 | -0,18 | -0,18 | 0,40 | 0,01 | -0,04 | -0,05 |  | 0,36 | 0,44 | 0,53 | 0,29 | 0,56 | 0,59 | 0,44 | -0,68 | -0,52 | -0,45 | -0,47 | -0,40 | -0,45 | -0,36 | -0,45 | -0,49 | -0,29 | -0,56 | 0,48 |
| **Attention Conners** | 0,06 | 0,17 | -0,16 | -0,16 | 0,21 | 0,10 | 0,07 | -0,29 | 0,36 |  | 0,50 | 0,30 | 0,36 | 0,73 | 0,33 | 0,13 | -0,27 | -0,05 | -0,35 | 0,00 | -0,09 | -0,19 | -0,16 | -0,11 | -0,16 | -0,33 | -0,27 | 0,33 |
| **Hyperactivity Conners** | -0,09 | 0,04 | 0,09 | 0,09 | 0,09 | -0,07 | 0,17 | 0,06 | 0,44 | 0,50 |  | 0,74 | 0,40 | 0,86 | 0,54 | 0,14 | -0,34 | -0,14 | -0,35 | -0,36 | 0,14 | -0,45 | -0,08 | -0,23 | -0,25 | -0,29 | -0,22 | 0,25 |
| **Impulsivity Conners** | -0,06 | -0,13 | 0,12 | 0,12 | 0,25 | -0,23 | 0,10 | -0,11 | 0,53 | 0,30 | 0,74 |  | 0,46 | 0,80 | 0,68 | 0,33 | -0,59 | -0,41 | -0,42 | -0,53 | -0,08 | -0,51 | -0,34 | -0,40 | -0,55 | -0,27 | -0,36 | 0,34 |
| **Self-Confidence Conners** | -0,30 | -0,20 | 0,11 | 0,11 | 0,00 | -0,17 | -0,07 | -0,25 | 0,29 | 0,36 | 0,40 | 0,46 |  | 0,67 | 0,39 | 0,35 | -0,29 | -0,05 | -0,37 | -0,11 | -0,13 | -0,34 | -0,04 | -0,21 | -0,32 | -0,15 | -0,19 | 0,12 |
| **Total Conners** | -0,07 | -0,02 | 0,01 | 0,01 | 0,20 | -0,13 | 0,09 | -0,19 | 0,56 | 0,73 | 0,86 | 0,80 | 0,67 |  | 0,60 | 0,29 | -0,47 | -0,18 | -0,49 | -0,29 | -0,08 | -0,49 | -0,22 | -0,24 | -0,42 | -0,32 | -0,33 | 0,37 |
| **BECK** | 0,08 | -0,12 | 0,09 | 0,09 | 0,19 | -0,02 | 0,26 | -0,35 | 0,59 | 0,33 | 0,54 | 0,68 | 0,39 | 0,60 |  | 0,55 | -0,68 | -0,36 | -0,60 | -0,50 | -0,19 | -0,66 | -0,31 | -0,43 | -0,64 | -0,33 | -0,33 | 0,30 |
| **ISI** | 0,10 | -0,06 | 0,23 | 0,24 | 0,14 | -0,23 | 0,19 | -0,16 | 0,44 | 0,13 | 0,14 | 0,33 | 0,35 | 0,29 | 0,55 |  | -0,48 | -0,38 | -0,31 | -0,36 | -0,17 | -0,28 | -0,23 | -0,44 | -0,51 | -0,19 | -0,29 | 0,03 |
| **General well being** | 0,04 | 0,24 | -0,09 | -0,09 | -0,45 | 0,01 | -0,08 | 0,22 | -0,68 | -0,27 | -0,34 | -0,59 | -0,29 | -0,47 | -0,68 | -0,48 |  | 0,72 | 0,71 | 0,67 | 0,47 | 0,70 | 0,71 | 0,58 | 0,81 | 0,48 | 0,60 | -0,53 |

Tables 2b. Determination matrix for adults. Each coefficient represents the coefficient of determination (**r²**) for two variables.

| **Determination coefficient** | **Age at diagnosis** | **Diagnostic delay** | **BMI** | **BMI z-score** | **AESS** | **Sleep latency** | **AHI** | **Sleep efficiency** | **Fatigue score** | **Attention Conners** | **Hyperactivity Conners** | **Impulsivity Conners** | **Self-Confidence Conners** | **Total Conners** | **BECK** | **ISI** | **General well being** | **Physical well being** | **Psychological well being** | **Physical activity** | **Vitality** | **psychological health** | **Physical limitation** | **Physical pain** | **Relations** | **Psychological limitation** | **Perceived health** | **Evolution of Perceived health** |
| --- | --- | --- | --- | --- | --- | --- | --- | --- | --- | --- | --- | --- | --- | --- | --- | --- | --- | --- | --- | --- | --- | --- | --- | --- | --- | --- | --- | --- |
| **Age at diagnosis** |  | 0,42 | 0,05 | 0,05 | 0,00 | 0,03 | 0,31 | 0,18 | 0,01 | 0,00 | 0,01 | 0,00 | 0,09 | 0,01 | 0,01 | 12,00 | 0,00 | 0,01 | 0,00 | 0,00 | 0,11 | 0,02 | 0,03 | 0,02 | 0,01 | 0,11 | 0,07 | 0,00 |
| **Diagnostic delay** | 0,42 |  | 0,00 | 0,00 | 0,02 | 0,00 | 0,30 | 0,09 | 0,06 | 0,03 | 0,00 | 0,02 | 0,04 | 0,00 | 0,02 | 0,00 | 0,06 | 0,02 | 0,04 | 0,01 | 0,09 | 0,05 | 0,01 | 0,03 | 0,07 | 0,00 | 0,02 | 0,05 |
| **BMI** | 0,05 | 0,00 |  | 1,00 | 0,02 | 0,01 | 0,09 | 0,00 | 0,03 | 0,03 | 0,01 | 0,01 | 0,01 | 0,00 | 0,01 | 0,06 | 0,01 | 0,01 | 0,00 | 0,02 | 0,01 | 0,00 | 0,00 | 0,01 | 0,03 | 0,00 | 0,00 | 0,02 |
| **BMI z-score** | 0,05 | 0,00 | 1,00 |  | 0,01 | 0,01 | 0,09 | 0,00 | 0,03 | 0,03 | 0,01 | 0,01 | 0,01 | 0,00 | 0,01 | 0,06 | 0,01 | 0,01 | 0,00 | 0,02 | 0,01 | 0,00 | 0,00 | 0,01 | 0,03 | 0,00 | 0,00 | 0,02 |
| **AESS** | 0,00 | 0,02 | 0,02 | 0,01 |  | 0,00 | 0,03 | 0,08 | 0,16 | 0,05 | 0,01 | 0,06 | 0,00 | 0,04 | 0,04 | 0,02 | 0,20 | 0,28 | 0,01 | 0,19 | 0,01 | 0,03 | 0,19 | 0,12 | 0,08 | 0,05 | 0,09 | 0,24 |
| **Sleep latency** | 0,03 | 0,00 | 0,01 | 0,01 | 0,00 |  | 0,02 | 0,00 | 0,00 | 0,01 | 0,00 | 0,05 | 0,03 | 0,02 | 0,00 | 0,05 | 0,00 | 0,02 | 0,02 | 0,00 | 0,01 | 0,00 | 0,01 | 0,03 | 0,05 | 0,02 | 0,03 | 0,01 |
| **AHI** | 0,31 | 0,30 | 0,09 | 0,09 | 0,03 | 0,02 |  | 0,13 | 0,00 | 0,00 | 0,03 | 0,01 | 0,00 | 0,01 | 0,07 | 0,04 | 0,01 | 0,02 | 0,00 | 0,09 | 0,11 | 0,00 | 0,03 | 0,00 | 0,01 | 0,07 | 0,00 | 0,02 |
| **Sleep efficiency** | 0,18 | 0,09 | 0,00 | 0,00 | 0,08 | 0,00 | 0,13 |  | 0,00 | 0,08 | 0,00 | 0,01 | 0,06 | 0,04 | 0,12 | 0,02 | 0,05 | 0,03 | 0,02 | 0,07 | 0,00 | 0,01 | 0,03 | 0,01 | 0,14 | 0,01 | 0,00 | 0,03 |
| **Fatigue score** | 0,01 | 0,06 | 0,03 | 0,03 | 0,16 | 0,00 | 0,00 | 0,00 |  | 0,13 | 0,19 | 0,28 | 0,09 | 0,31 | 0,35 | 0,19 | 0,46 | 0,27 | 0,20 | 0,22 | 0,16 | 0,21 | 0,13 | 0,20 | 0,24 | 0,08 | 0,31 | 0,23 |
| **Attention Conners** | 0,00 | 0,03 | 0,03 | 0,03 | 0,05 | 0,01 | 0,00 | 0,08 | 0,13 |  | 0,25 | 0,09 | 0,13 | 0,53 | 0,11 | 0,02 | 0,08 | 0,00 | 0,12 | 0,00 | 0,01 | 0,04 | 0,03 | 0,01 | 0,02 | 0,11 | 0,07 | 0,11 |
| **Hyperactivity Conners** | 0,01 | 0,00 | 0,01 | 0,01 | 0,01 | 0,00 | 0,03 | 0,00 | 0,19 | 0,25 |  | 0,55 | 0,16 | 0,73 | 0,29 | 0,02 | 0,12 | 0,02 | 0,12 | 0,13 | 0,02 | 0,20 | 0,01 | 0,05 | 0,06 | 0,09 | 0,05 | 0,06 |
| **Impulsivity Conners** | 0,00 | 0,02 | 0,01 | 0,01 | 0,06 | 0,05 | 0,01 | 0,01 | 0,28 | 0,09 | 0,55 |  | 0,21 | 0,64 | 0,46 | 0,11 | 0,35 | 0,17 | 0,17 | 0,28 | 0,01 | 0,26 | 0,11 | 0,16 | 0,30 | 0,07 | 0,13 | 0,12 |
| **Self-Confidence Conners** | 0,09 | 0,04 | 0,01 | 0,01 | 0,00 | 0,03 | 0,00 | 0,06 | 0,09 | 0,13 | 0,16 | 0,21 |  | 0,45 | 0,15 | 0,12 | 0,09 | 0,00 | 0,13 | 0,01 | 0,02 | 0,12 | 0,00 | 0,05 | 0,10 | 0,02 | 0,03 | 0,01 |
| **Total Conners** | 0,01 | 0,00 | 0,00 | 0,00 | 0,04 | 0,02 | 0,01 | 0,04 | 0,31 | 0,53 | 0,73 | 0,64 | 0,45 |  | 0,36 | 0,09 | 0,22 | 0,03 | 0,24 | 0,08 | 0,01 | 0,24 | 0,05 | 0,06 | 0,18 | 0,10 | 0,11 | 0,14 |
| **BECK** | 0,01 | 0,02 | 0,01 | 0,01 | 0,04 | 0,00 | 0,07 | 0,12 | 0,35 | 0,11 | 0,29 | 0,46 | 0,15 | 0,36 |  | 0,30 | 0,46 | 0,13 | 0,36 | 0,25 | 0,04 | 0,44 | 0,10 | 0,19 | 0,41 | 0,11 | 0,11 | 0,09 |
| **ISI** | 0,01 | 0,00 | 0,06 | 0,06 | 0,02 | 0,05 | 0,04 | 0,02 | 0,19 | 0,02 | 0,02 | 0,11 | 0,12 | 0,09 | 0,30 |  | 0,23 | 0,14 | 0,10 | 0,13 | 0,03 | 0,08 | 0,05 | 0,20 | 0,26 | 0,04 | 0,09 | 0,00 |
| **General well being** | 0,00 | 0,06 | 0,01 | 0,01 | 0,20 | 0,00 | 0,01 | 0,05 | 0,46 | 0,08 | 0,12 | 0,35 | 0,09 | 0,22 | 0,46 | 0,23 |  | 0,52 | 0,51 | 0,45 | 0,22 | 0,50 | 0,51 | 0,34 | 0,66 | 0,23 | 0,37 | 0,28 |

Red cells are significant, beige cells are trend.

Tables 2c. Number of cases (N) matrix for adults

| **N** | **Age at diagnosis** | **Diagnostic delay** | **BMI** | **BMI z-score** | **AESS** | **Sleep latency** | **AHI** | **Sleep efficiency** | **Fatigue score** | **Attention Conners** | **Hyperactivity Conners** | **Impulsivity Conners** | **Self-Confidence Conners** | **Total Conners** | **BECK** | **ISI** | **General well being** | **Physical well being** | **Psychological well being** | **Physical activity** | **Vitality** | **psychological health** | **Physical limitation** | **Physical pain** | **Relations** | **Psychological limitation** | **Perceived health** | **Evolution of Perceived health** |
| --- | --- | --- | --- | --- | --- | --- | --- | --- | --- | --- | --- | --- | --- | --- | --- | --- | --- | --- | --- | --- | --- | --- | --- | --- | --- | --- | --- | --- |
| **Age at diagnosis** | 46 | 46 | 46 | 46 | 42 | 46 | 45 | 44 | 38 | 38 | 38 | 37 | 38 | 37 | 38 | 37 | 39 | 39 | 39 | 39 | 39 | 39 | 39 | 39 | 39 | 39 | 39 | 39 |
| **Diagnostic delay** | 46 | 46 | 46 | 46 | 42 | 46 | 45 | 44 | 38 | 38 | 38 | 37 | 38 | 37 | 38 | 37 | 39 | 39 | 39 | 39 | 39 | 39 | 39 | 39 | 39 | 39 | 39 | 39 |
| **BMI** | 46 | 46 | 46 | 46 | 42 | 46 | 45 | 44 | 38 | 38 | 38 | 37 | 38 | 37 | 38 | 37 | 39 | 39 | 39 | 39 | 39 | 39 | 39 | 39 | 39 | 39 | 39 | 39 |
| **BMI z-score** | 46 | 46 | 46 | 46 | 42 | 46 | 45 | 44 | 38 | 38 | 38 | 37 | 38 | 37 | 38 | 37 | 39 | 39 | 39 | 39 | 39 | 39 | 39 | 39 | 39 | 39 | 39 | 39 |
| **AESS** | 42 | 42 | 42 | 42 | 42 | 42 | 41 | 40 | 38 | 38 | 38 | 37 | 38 | 37 | 38 | 37 | 39 | 39 | 39 | 39 | 39 | 39 | 39 | 39 | 39 | 39 | 39 | 39 |
| **Sleep latency** | 46 | 46 | 46 | 46 | 42 | 46 | 45 | 44 | 38 | 38 | 38 | 37 | 38 | 37 | 38 | 37 | 39 | 39 | 39 | 39 | 39 | 39 | 39 | 39 | 39 | 39 | 39 | 39 |
| **AHI** | 45 | 45 | 45 | 45 | 41 | 45 | 45 | 43 | 37 | 37 | 37 | 36 | 37 | 36 | 37 | 36 | 38 | 38 | 38 | 38 | 38 | 38 | 38 | 38 | 38 | 38 | 38 | 38 |
| **Sleep efficiency** | 44 | 44 | 44 | 44 | 40 | 44 | 43 | 44 | 36 | 37 | 37 | 36 | 37 | 36 | 37 | 36 | 38 | 38 | 38 | 38 | 38 | 38 | 38 | 38 | 38 | 38 | 38 | 38 |
| **Fatigue score** | 38 | 38 | 38 | 38 | 38 | 38 | 37 | 36 | 38 | 36 | 36 | 35 | 36 | 35 | 36 | 36 | 37 | 37 | 37 | 37 | 37 | 37 | 37 | 37 | 37 | 37 | 37 | 37 |
| **Attention Conners** | 38 | 38 | 38 | 38 | 38 | 38 | 37 | 37 | 36 | 38 | 38 | 37 | 38 | 37 | 37 | 36 | 38 | 38 | 38 | 38 | 38 | 38 | 38 | 38 | 38 | 38 | 38 | 38 |
| **Hyperactivity Conners** | 38 | 38 | 38 | 38 | 38 | 38 | 37 | 37 | 36 | 38 | 38 | 37 | 38 | 37 | 37 | 36 | 38 | 38 | 38 | 38 | 38 | 38 | 38 | 38 | 38 | 38 | 38 | 38 |
| **Impulsivity Conners** | 37 | 37 | 37 | 37 | 37 | 37 | 36 | 36 | 35 | 37 | 37 | 37 | 37 | 37 | 36 | 35 | 37 | 37 | 37 | 37 | 37 | 37 | 37 | 37 | 37 | 37 | 37 | 37 |
| **Self-Confidence Conners** | 38 | 38 | 38 | 38 | 38 | 38 | 37 | 37 | 36 | 38 | 38 | 37 | 38 | 37 | 37 | 36 | 38 | 38 | 38 | 38 | 38 | 38 | 38 | 38 | 38 | 38 | 38 | 38 |
| **Total Conners** | 37 | 37 | 37 | 37 | 37 | 37 | 36 | 36 | 35 | 37 | 37 | 37 | 37 | 37 | 36 | 35 | 37 | 37 | 37 | 37 | 37 | 37 | 37 | 37 | 37 | 37 | 37 | 37 |
| **BECK** | 38 | 38 | 38 | 38 | 38 | 38 | 37 | 37 | 36 | 37 | 37 | 36 | 37 | 36 | 38 | 36 | 38 | 38 | 38 | 38 | 38 | 38 | 38 | 38 | 38 | 38 | 38 | 38 |
| **ISI** | 37 | 37 | 37 | 37 | 37 | 37 | 36 | 36 | 36 | 36 | 36 | 35 | 36 | 35 | 36 | 37 | 37 | 37 | 37 | 37 | 37 | 37 | 37 | 37 | 37 | 37 | 37 | 37 |
| **General well being** | 39 | 39 | 39 | 39 | 39 | 39 | 38 | 38 | 37 | 38 | 38 | 37 | 38 | 37 | 38 | 37 | 39 | 39 | 39 | 39 | 39 | 39 | 39 | 39 | 39 | 39 | 39 | 39 |
|  |  |  |  |  |  |  |  |  |  |  |  |  |  |  |  |  |  |  |  |  |  |  |  |  |  |  |  |  |
| ***p*** | **Age at diagnosis** | **Diagnostic delay** | **BMI** | **BMI z-score** | **AESS** | **Sleep latency** | **AHI** | **Sleep efficiency** | **Fatigue score** | **Attention Conners** | **Hyperactivity Conners** | **Impulsivity Conners** | **Self-Confidence Conners** | **Total Conners** | **BECK** | **ISI** | **General well being** | **Physical well being** | **Psychological well being** | **Physical activity** | **Vitality** | **psychological health** | **Physical limitation** | **Physical pain** | **Relations** | **Psychological limitation** | **Perceived health** | **Evolution of Perceived health** |
| **Age at diagnosis** |  | 0,00 | 0,15 | 0,15 | 0,71 | 0,28 | 0,00 | 0,00 | 0,57 | 0,74 | 0,61 | 0,72 | 0,07 | 0,67 | 0,63 | 0,57 | 0,80 | 0,47 | 0,71 | 0,89 | 0,04 | 0,41 | 0,31 | 0,35 | 0,65 | 0,04 | 0,11 | 0,98 |
| **Diagnostic delay** | 0,00 |  | 0,80 | 0,79 | 0,35 | 0,66 | 0,00 | 0,05 | 0,15 | 0,32 | 0,83 | 0,45 | 0,22 | 0,92 | 0,46 | 0,74 | 0,15 | 0,43 | 0,21 | 0,62 | 0,06 | 0,17 | 0,67 | 0,27 | 0,10 | 0,73 | 0,35 | 0,15 |
| **BMI** | 0,15 | 0,80 |  | 0,00 | 0,44 | 0,61 | 0,05 | 0,79 | 0,28 | 0,34 | 0,59 | 0,49 | 0,52 | 0,93 | 0,58 | 0,16 | 0,58 | 0,54 | 0,85 | 0,36 | 0,60 | 0,93 | 0,80 | 0,63 | 0,29 | 0,67 | 0,78 | 0,43 |
| **BMI z-score** | 0,15 | 0,79 | 0,00 |  | 0,45 | 0,59 | 0,05 | 0,81 | 0,28 | 0,33 | 0,60 | 0,49 | 0,52 | 0,94 | 0,58 | 0,16 | 0,57 | 0,53 | 0,85 | 0,36 | 0,63 | 0,92 | 0,79 | 0,62 | 0,29 | 0,70 | 0,77 | 0,42 |
| **AESS** | 0,71 | 0,35 | 0,44 | 0,45 |  | 0,75 | 0,25 | 0,07 | 0,01 | 0,20 | 0,59 | 0,14 | 0,99 | 0,23 | 0,25 | 0,40 | 0,00 | 0,00 | 0,48 | 0,01 | 0,66 | 0,26 | 0,01 | 0,03 | 0,08 | 0,18 | 0,06 | 0,00 |
| **Sleep latency** | 0,28 | 0,66 | 0,61 | 0,59 | 0,75 |  | 0,40 | 0,75 | 0,94 | 0,54 | 0,70 | 0,18 | 0,30 | 0,46 | 0,92 | 0,17 | 0,97 | 0,41 | 0,37 | 0,99 | 0,50 | 0,98 | 0,56 | 0,30 | 0,16 | 0,38 | 0,26 | 0,54 |
| **AHI** | 0,00 | 0,00 | 0,05 | 0,05 | 0,25 | 0,40 |  | 0,02 | 0,81 | 0,69 | 0,32 | 0,58 | 0,69 | 0,62 | 0,13 | 0,26 | 0,65 | 0,41 | 0,86 | 0,07 | 0,04 | 0,71 | 0,34 | 0,81 | 0,62 | 0,10 | 0,92 | 0,40 |
| **Sleep efficiency** | 0,00 | 0,05 | 0,79 | 0,81 | 0,07 | 0,75 | 0,02 |  | 0,79 | 0,08 | 0,70 | 0,51 | 0,13 | 0,27 | 0,03 | 0,36 | 0,19 | 0,28 | 0,43 | 0,11 | 0,96 | 0,63 | 0,29 | 0,61 | 0,02 | 0,48 | 0,78 | 0,29 |
| **Fatigue score** | 0,57 | 0,15 | 0,28 | 0,28 | 0,01 | 0,94 | 0,81 | 0,79 |  | 0,03 | 0,01 | 0,00 | 0,08 | 0,00 | 0,00 | 0,01 | 0,00 | 0,00 | 0,00 | 0,00 | 0,01 | 0,00 | 0,03 | 0,01 | 0,00 | 0,09 | 0,00 | 0,00 |
| **Attention Conners** | 0,74 | 0,32 | 0,34 | 0,33 | 0,20 | 0,54 | 0,69 | 0,08 | 0,03 |  | 0,00 | 0,07 | 0,03 | 0,00 | 0,04 | 0,46 | 0,10 | 0,78 | 0,03 | 0,99 | 0,61 | 0,26 | 0,34 | 0,51 | 0,35 | 0,04 | 0,10 | 0,04 |
| **Hyperactivity Conners** | 0,61 | 0,83 | 0,59 | 0,60 | 0,59 | 0,70 | 0,32 | 0,70 | 0,01 | 0,00 |  | 0,00 | 0,01 | 0,00 | 0,00 | 0,43 | 0,04 | 0,39 | 0,03 | 0,03 | 0,40 | 0,00 | 0,64 | 0,17 | 0,13 | 0,07 | 0,19 | 0,13 |
| **Impulsivity Conners** | 0,72 | 0,45 | 0,49 | 0,49 | 0,14 | 0,18 | 0,58 | 0,51 | 0,00 | 0,07 | 0,00 |  | 0,00 | 0,00 | 0,00 | 0,05 | 0,00 | 0,01 | 0,01 | 0,00 | 0,63 | 0,00 | 0,04 | 0,01 | 0,00 | 0,10 | 0,03 | 0,04 |
| **Self-Confidence Conners** | 0,07 | 0,22 | 0,52 | 0,52 | 0,99 | 0,30 | 0,69 | 0,13 | 0,08 | 0,03 | 0,01 | 0,00 |  | 0,00 | 0,02 | 0,04 | 0,07 | 0,74 | 0,02 | 0,49 | 0,42 | 0,04 | 0,79 | 0,20 | 0,05 | 0,36 | 0,26 | 0,48 |
| **Total Conners** | 0,67 | 0,92 | 0,93 | 0,94 | 0,23 | 0,46 | 0,62 | 0,27 | 0,00 | 0,00 | 0,00 | 0,00 | 0,00 |  | 0,00 | 0,09 | 0,00 | 0,29 | 0,00 | 0,09 | 0,66 | 0,00 | 0,19 | 0,16 | 0,01 | 0,05 | 0,04 | 0,02 |
| **BECK** | 0,63 | 0,46 | 0,58 | 0,58 | 0,25 | 0,92 | 0,13 | 0,03 | 0,00 | 0,04 | 0,00 | 0,00 | 0,02 | 0,00 |  | 0,00 | 0,00 | 0,03 | 0,00 | 0,00 | 0,26 | 0,00 | 0,05 | 0,01 | 0,00 | 0,04 | 0,04 | 0,07 |
| **ISI** | 0,57 | 0,74 | 0,16 | 0,16 | 0,40 | 0,17 | 0,26 | 0,36 | 0,01 | 0,46 | 0,43 | 0,05 | 0,04 | 0,09 | 0,00 |  | 0,00 | 0,02 | 0,06 | 0,03 | 0,32 | 0,09 | 0,17 | 0,01 | 0,00 | 0,25 | 0,08 | 0,87 |
| **General well being** | 0,80 | 0,15 | 0,58 | 0,57 | 0,00 | 0,97 | 0,65 | 0,19 | 0,00 | 0,10 | 0,04 | 0,00 | 0,07 | 0,00 | 0,00 | 0,00 |  | 0,00 | 0,00 | 0,00 | 0,00 | 0,00 | 0,00 | 0,00 | 0,00 | 0,00 | 0,00 | 0,00 |

Tables 2d. p-value matrix for adults

Supplementary material 3

Table 3a. Pearson correlation matrix for children. Each coefficient represents the linear correlation (**r**) between two variables.

| **Correlation coefficient** | **Age at diagnosis** | **Diagnostic delay** | **BMI** | **BMI z-score** | **AESS** | **Sleep latency** | **AHI** | **Sleep efficiency** | **Fatigue score** | **Impulsivity Conners** | **Total Conners** | **ISI** | **CDI** | **General well being** | **Physical activity** | **Vitality** | **psychological health** |
| --- | --- | --- | --- | --- | --- | --- | --- | --- | --- | --- | --- | --- | --- | --- | --- | --- | --- |
| **Age at diagnosis** | 1,00 | 0,40 | 0,16 | -0,29 | -0,29 | -0,13 | -0,01 | 0,34 | 0,09 | 0,16 | -0,04 | 0,01 | 0,12 | -0,12 | 0,22 | 0,02 | 0,17 |
| **Diagnostic delay** | 0,40 | 1,00 | 0,25 | 0,03 | -0,04 | -0,09 | -0,13 | -0,06 | 0,41 | -0,01 | -0,05 | 0,10 | 0,37 | 0,08 | -0,02 | 0,13 | 0,16 |
| **BMI** | 0,16 | 0,25 | 1,00 | 0,87 | 0,04 | -0,02 | -0,03 | -0,03 | 0,18 | -0,13 | -0,03 | 0,23 | 0,15 | -0,30 | 0,06 | -0,24 | -0,23 |
| **BMI z-score** | -0,29 | 0,03 | 0,87 | 1,00 | 0,14 | 0,03 | -0,04 | -0,18 | 0,07 | -0,24 | -0,02 | 0,23 | 0,04 | -0,22 | 0,00 | -0,26 | -0,32 |
| **AESS** | -0,29 | -0,04 | 0,04 | 0,14 | 1,00 | 0,19 | -0,18 | -0,37 | 0,38 | 0,13 | 0,25 | 0,05 | 0,06 | 0,02 | -0,27 | 0,05 | -0,11 |
| **Sleep latency** | -0,13 | -0,09 | -0,02 | 0,03 | 0,19 | 1,00 | 0,13 | -0,24 | 0,04 | 0,17 | -0,08 | 0,10 | -0,04 | -0,27 | -0,24 | -0,19 | -0,14 |
| **AHI** | -0,01 | -0,13 | -0,03 | -0,04 | -0,18 | 0,13 | 1,00 | 0,02 | 0,03 | -0,16 | -0,11 | 0,19 | -0,17 | -0,02 | 0,01 | 0,01 | 0,15 |
| **Sleep efficiency** | 0,34 | -0,06 | -0,03 | -0,18 | -0,37 | -0,24 | 0,02 | 1,00 | -0,23 | -0,02 | -0,07 | -0,05 | 0,06 | -0,04 | 0,12 | 0,01 | 0,06 |
| **Fatigue score** | 0,09 | 0,41 | 0,18 | 0,07 | 0,38 | 0,04 | 0,03 | -0,23 | 1,00 | 0,29 | 0,59 | 0,55 | 0,63 | -0,29 | -0,50 | -0,17 | -0,21 |
| **Impulsivity Conners** | 0,16 | -0,01 | -0,13 | -0,24 | 0,13 | 0,17 | -0,16 | -0,02 | 0,29 | 1,00 | 0,55 | 0,10 | 0,31 | -0,37 | -0,51 | -0,25 | -0,29 |
| **Total Conners** | -0,04 | -0,05 | -0,03 | -0,02 | 0,25 | -0,08 | -0,11 | -0,07 | 0,59 | 0,55 | 1,00 | 0,37 | 0,47 | -0,41 | -0,51 | -0,45 | -0,50 |
| **ISI** | 0,01 | 0,10 | 0,23 | 0,23 | 0,05 | 0,10 | 0,19 | -0,05 | 0,55 | 0,10 | 0,37 | 1,00 | 0,47 | -0,55 | -0,46 | -0,52 | -0,68 |
| **CDI** | 0,12 | 0,37 | 0,15 | 0,04 | 0,06 | -0,04 | -0,17 | 0,06 | 0,63 | 0,31 | 0,47 | 0,47 | 1,00 | -0,67 | -0,51 | -0,60 | -0,50 |
| **General well being** | -0,12 | 0,08 | -0,30 | -0,22 | 0,02 | -0,27 | -0,02 | -0,04 | -0,29 | -0,37 | -0,41 | -0,55 | -0,67 | 1,00 | 0,56 | 0,93 | 0,82 |

Table 3b. Determination matrix for children. Each coefficient represents the coefficient of determination (**r²**) for two variables.

| **Determination coefficient** | **Age at diagnosis** | **Diagnostic delay** | **BMI** | **BMI z-score** | **AESS** | **Sleep latency** | **AHI** | **Sleep efficiency** | **Fatigue score** | **Impulsivity Conners** | **Total Conners** | **ISI** | **CDI** | **General well being** | **Physical activity** | **Vitality** | **psychological health** |
| --- | --- | --- | --- | --- | --- | --- | --- | --- | --- | --- | --- | --- | --- | --- | --- | --- | --- |
| **Age at diagnosis** | 1,00 | 0,16 | 0,03 | 0,09 | 0,08 | 0,02 | 0,00 | 0,12 | 0,01 | 0,03 | 0,00 | 0,00 | 0,01 | 0,01 | 0,05 | 0,00 | 0,03 |
| **Diagnostic delay** | 0,16 | 1,00 | 0,06 | 0,00 | 0,00 | 0,01 | 0,02 | 0,00 | 0,17 | 0,00 | 0,00 | 0,01 | 0,14 | 0,01 | 0,00 | 0,02 | 0,03 |
| **BMI** | 0,03 | 0,06 | 1,00 | 0,77 | 0,00 | 0,00 | 0,00 | 0,00 | 0,03 | 0,02 | 0,00 | 0,05 | 0,02 | 0,09 | 0,00 | 0,06 | 0,06 |
| **BMI z-score** | 0,09 | 0,00 | 0,77 | 1,00 | 0,02 | 0,00 | 0,00 | 0,03 | 0,00 | 0,06 | 0,00 | 0,05 | 0,00 | 0,05 | 0,00 | 0,07 | 0,10 |
| **AESS** | 0,08 | 0,00 | 0,00 | 0,02 | 1,00 | 0,04 | 0,03 | 0,14 | 0,14 | 0,02 | 0,06 | 0,00 | 0,00 | 0,00 | 0,07 | 0,00 | 0,01 |
| **Sleep latency** | 0,02 | 0,01 | 0,00 | 0,00 | 0,04 | 1,00 | 0,02 | 0,06 | 0,00 | 0,03 | 0,01 | 0,01 | 0,00 | 0,07 | 0,06 | 0,04 | 0,02 |
| **AHI** | 0,00 | 0,02 | 0,00 | 0,00 | 0,03 | 0,02 | 1,00 | 0,00 | 0,00 | 0,03 | 0,01 | 0,04 | 0,03 | 0,00 | 0,00 | 0,00 | 0,02 |
| **Sleep efficiency** | 0,12 | 0,00 | 0,00 | 0,03 | 0,14 | 0,06 | 0,00 | 1,00 | 0,05 | 0,00 | 0,01 | 0,00 | 0,00 | 0,00 | 0,01 | 0,00 | 0,00 |
| **Fatigue score** | 0,01 | 0,17 | 0,03 | 0,00 | 0,14 | 0,00 | 0,00 | 0,05 | 1,00 | 0,09 | 0,35 | 0,30 | 0,39 | 0,08 | 0,25 | 0,03 | 0,04 |
| **Impulsivity Conners** | 0,03 | 0,00 | 0,02 | 0,06 | 0,02 | 0,03 | 0,03 | 0,00 | 0,09 | 1,00 | 0,31 | 0,01 | 0,09 | 0,14 | 0,26 | 0,06 | 0,08 |
| **Total Conners** | 0,00 | 0,00 | 0,00 | 0,00 | 0,06 | 0,01 | 0,01 | 0,01 | 0,35 | 0,31 | 1,00 | 0,14 | 0,22 | 0,17 | 0,26 | 0,21 | 0,25 |
| **ISI** | 0,00 | 0,01 | 0,05 | 0,05 | 0,00 | 0,01 | 0,04 | 0,00 | 0,30 | 0,01 | 0,14 | 1,00 | 0,22 | 0,30 | 0,21 | 0,27 | 0,47 |
| **CDI** | 0,01 | 0,14 | 0,02 | 0,00 | 0,00 | 0,00 | 0,03 | 0,00 | 0,39 | 0,09 | 0,22 | 0,22 | 1,00 | 0,44 | 0,26 | 0,36 | 0,25 |
| **General well being** | 0,01 | 0,01 | 0,09 | 0,05 | 0,00 | 0,07 | 0,00 | 0,00 | 0,08 | 0,14 | 0,17 | 0,30 | 0,44 | 1,00 | 0,31 | 0,87 | 0,67 |

Table 3c. Number of cases (N) matrix for children.

| N | **Age at diagnosis** | **Diagnostic delay** | **BMI** | **BMI z-score** | **AESS** | **Sleep latency** | **AHI** | **Sleep efficiency** | **Fatigue score** | **Impulsivity Conners** | **Total Conners** | **ISI** | **CDI** | **General well being** | **Physical activity** | **Vitality** | **psychological health** |
| --- | --- | --- | --- | --- | --- | --- | --- | --- | --- | --- | --- | --- | --- | --- | --- | --- | --- |
| Age at diagnosis | 46 | 46 | 46 | 46 | 37 | 39 | 39 | 39 | 32 | 29 | 29 | 33 | 33 | 27 | 29 | 29 | 29 |
| Diagnostic delay | 46 | 46 | 46 | 46 | 37 | 39 | 39 | 39 | 32 | 29 | 29 | 33 | 33 | 27 | 29 | 29 | 29 |
| BMI | 46 | 46 | 46 | 46 | 37 | 39 | 39 | 39 | 32 | 29 | 29 | 33 | 33 | 27 | 29 | 29 | 29 |
| BMI z-score | 46 | 46 | 46 | 46 | 37 | 39 | 39 | 39 | 32 | 29 | 29 | 33 | 33 | 27 | 29 | 29 | 29 |
| AESS | 37 | 37 | 37 | 37 | 37 | 30 | 30 | 30 | 27 | 23 | 23 | 27 | 27 | 23 | 24 | 24 | 24 |
| Sleep latency | 39 | 39 | 39 | 39 | 30 | 39 | 39 | 39 | 31 | 27 | 27 | 32 | 32 | 27 | 29 | 29 | 29 |
| AHI | 39 | 39 | 39 | 39 | 30 | 39 | 39 | 39 | 31 | 27 | 27 | 32 | 32 | 27 | 29 | 29 | 29 |
| Sleep efficiency | 39 | 39 | 39 | 39 | 30 | 39 | 39 | 39 | 31 | 27 | 27 | 32 | 32 | 27 | 29 | 29 | 29 |
| Fatigue score | 32 | 32 | 32 | 32 | 27 | 31 | 31 | 31 | 32 | 27 | 27 | 32 | 32 | 26 | 28 | 28 | 28 |
| Impulsivity Conners | 29 | 29 | 29 | 29 | 23 | 27 | 27 | 27 | 27 | 29 | 29 | 28 | 28 | 23 | 25 | 25 | 25 |
| Total Conners | 29 | 29 | 29 | 29 | 23 | 27 | 27 | 27 | 27 | 29 | 29 | 28 | 28 | 23 | 25 | 25 | 25 |
| ISI | 33 | 33 | 33 | 33 | 27 | 32 | 32 | 32 | 32 | 28 | 28 | 33 | 33 | 27 | 29 | 29 | 29 |
| CDI | 33 | 33 | 33 | 33 | 27 | 32 | 32 | 32 | 32 | 28 | 28 | 33 | 33 | 27 | 29 | 29 | 29 |
| General well being | 27 | 27 | 27 | 27 | 23 | 27 | 27 | 27 | 26 | 23 | 23 | 27 | 27 | 27 | 27 | 27 | 27 |

Table 3d. p-value matrix for children.

| Significance (p) | **Age at diagnosis** | **Diagnostic delay** | **BMI** | **BMI z-score** | **AESS** | **Sleep latency** | **AHI** | **Sleep efficiency** | **Fatigue score** | **Impulsivity Conners** | **Total Conners** | **ISI** | **CDI** | **General well being** | **Physical activity** | **Vitality** | **psychological health** |
| --- | --- | --- | --- | --- | --- | --- | --- | --- | --- | --- | --- | --- | --- | --- | --- | --- | --- |
| Age at diagnosis |  | 0,005609 | 0,276662 | 0,048491 | 0,081496 | 0,414919 | 0,972053 | 0,032861 | 0,614161 | 0,401786 | 0,849698 | 0,962748 | 0,501744 | 0,550241 | 0,244706 | 0,901983 | 0,384379 |
| Diagnostic delay | 0,005609 |  | 0,08757 | 0,843311 | 0,804378 | 0,58173 | 0,428037 | 0,738972 | 0,019745 | 0,949607 | 0,801732 | 0,566461 | 0,031611 | 0,680173 | 0,906328 | 0,514546 | 0,394072 |
| BMI | 0,276662 | 0,08757 |  | 1,78E-15 | 0,818616 | 0,890471 | 0,879442 | 0,84123 | 0,336322 | 0,517651 | 0,897192 | 0,195603 | 0,411908 | 0,131879 | 0,771039 | 0,21015 | 0,220301 |
| BMI z-score | 0,048491 | 0,843311 | 1,78E-15 |  | 0,418996 | 0,879456 | 0,803983 | 0,272904 | 0,704022 | 0,219762 | 0,929014 | 0,206915 | 0,825736 | 0,259661 | 0,993021 | 0,172004 | 0,092661 |
| AESS | 0,081496 | 0,804378 | 0,818616 | 0,418996 |  | 0,302102 | 0,349353 | 0,041346 | 0,052853 | 0,559564 | 0,247127 | 0,818238 | 0,769097 | 0,943198 | 0,200096 | 0,803462 | 0,600995 |
| Sleep latency | 0,414919 | 0,58173 | 0,890471 | 0,879456 | 0,302102 |  | 0,419412 | 0,138185 | 0,816761 | 0,405201 | 0,696636 | 0,573033 | 0,830101 | 0,169688 | 0,211017 | 0,313924 | 0,481202 |
| AHI | 0,972053 | 0,428037 | 0,879442 | 0,803983 | 0,349353 | 0,419412 |  | 0,909145 | 0,891929 | 0,42517 | 0,578326 | 0,284995 | 0,361939 | 0,923324 | 0,959596 | 0,95306 | 0,447222 |
| Sleep efficiency | 0,032861 | 0,738972 | 0,84123 | 0,272904 | 0,041346 | 0,138185 | 0,909145 |  | 0,214597 | 0,918529 | 0,722856 | 0,80299 | 0,750081 | 0,854226 | 0,53167 | 0,943881 | 0,764617 |
| Fatigue score | 0,614161 | 0,019745 | 0,336322 | 0,704022 | 0,052853 | 0,816761 | 0,891929 | 0,214597 |  | 0,138922 | 0,001155 | 0,001204 | 0,000124 | 0,151365 | 0,006652 | 0,389317 | 0,279719 |
| Impulsivity Conners | 0,401786 | 0,949607 | 0,517651 | 0,219762 | 0,559564 | 0,405201 | 0,42517 | 0,918529 | 0,138922 |  | 0,001876 | 0,614549 | 0,112815 | 0,082626 | 0,009449 | 0,233696 | 0,158958 |
| Total Conners | 0,849698 | 0,801732 | 0,897192 | 0,929014 | 0,247127 | 0,696636 | 0,578326 | 0,722856 | 0,001155 | 0,001876 |  | 0,050081 | 0,011342 | 0,052601 | 0,008906 | 0,022933 | 0,010652 |
| ISI | 0,962748 | 0,566461 | 0,195603 | 0,206915 | 0,818238 | 0,573033 | 0,284995 | 0,80299 | 0,001204 | 0,614549 | 0,050081 |  | 0,005711 | 0,003256 | 0,011302 | 0,004092 | 4,42E-05 |
| CDI | 0,501744 | 0,031611 | 0,411908 | 0,825736 | 0,769097 | 0,830101 | 0,361939 | 0,750081 | 0,000124 | 0,112815 | 0,011342 | 0,005711 |  | 0,00015 | 0,004475 | 0,000548 | 0,005345 |
| General well being | 0,550241 | 0,680173 | 0,131879 | 0,259661 | 0,943198 | 0,169688 | 0,923324 | 0,854226 | 0,151365 | 0,082626 | 0,052601 | 0,003256 | 0,00015 |  | 0,002617 | 1,53E-12 | 2,15E-07 |

Red cells are significant, beige cells are trend.
